# RNA silencing is a key regulatory mechanism in the biocontrol fungus *Clonostachys rosea*-wheat interactions

**DOI:** 10.1186/s12915-024-02014-9

**Published:** 2024-09-30

**Authors:** Edoardo Piombo, Ramesh Raju Vetukuri, Naga Charan Konakalla, Pruthvi B. Kalyandurg, Poorva Sundararajan, Dan Funck Jensen, Magnus Karlsson, Mukesh Dubey

**Affiliations:** 1 Department of Forest Mycology and Plant Pathology, Swedish University of Agricultural Sciences, Uppsala, Sweden; 2 Department of Plant Breeding, Swedish University of Agricultural Sciences, Lomma, Sweden

**Keywords:** Beneficial fungi, Cross-kingdom RNA silencing, DCL, Defense induction, Gene silencing, Growth promotion, miRNA, RNA interference, sRNAs, *Triticum aestivum*

## Abstract

**Background:**

Small RNA (sRNAs)- mediated RNA silencing is emerging as a key player in host-microbe interactions. However, its role in fungus-plant interactions relevant to biocontrol of plant diseases is yet to be explored. This study aimed to investigate Dicer (DCL)-mediated endogenous and cross-kingdom gene expression regulation in the biocontrol fungus *Clonostachys rosea* and wheat roots during interactions.

**Results:**

*C. rosea* Δ*dcl2* strain exhibited significantly higher root colonization than the WT, whereas no significant differences were observed for Δ*dcl1* strains. Dual RNA-seq revealed the upregulation of CAZymes, membrane transporters, and effector coding genes in *C. rosea*, whereas wheat roots responded with the upregulation of stress-related genes and the downregulation of growth-related genes. The expression of many of these genes was downregulated in wheat during the interaction with DCL deletion strains, underscoring the influence of fungal DCL genes on wheat defense response. sRNA sequencing identified 18 wheat miRNAs responsive to *C. rosea*, and three were predicted to target the *C. rosea* polyketide synthase gene *pks29*. Two of these miRNAs (mir_17532_x1 and mir_12061_x13) were observed to enter *C. rosea* from wheat roots with fluorescence analyses and to downregulate the expression of *pks29*, showing plausible cross-kingdom RNA silencing of the *C. rosea* gene by wheat miRNAs.

**Conclusions:**

We provide insights into the mechanisms underlying the interaction between biocontrol fungi and plant roots. Moreover, the study sheds light on the role of sRNA-mediated gene expression regulation in *C. rosea*-wheat interactions and provides preliminary evidence of cross-kingdom RNA silencing between plants and biocontrol fungi.

**Supplementary Information:**

The online version contains supplementary material available at 10.1186/s12915-024-02014-9.

## Background

The genetic information flows from DNA to RNA to protein via transcription and translation, respectively. This flow of information is regulated at the transcriptional and post-transcriptional levels to maintain the proper functioning of cells. Post-transcriptional gene silencing (PTGS) is a highly conserved process of gene expression regulation, also called RNA silencing. This activity is performed by small non-coding RNAs (sRNAs) commonly ranging from 18 to 40 nucleotides (nt) in size [1–3]. The most studied sRNAs are typically produced from double-stranded RNAs and single-stranded RNAs with stem-loop structures. This is carried out through enzymatic cleavage by endoribonuclease called Dicer (or Dicer-like [DCL] in fungi), producing small interfering RNAs (siRNAs) and microRNAs (miRNAs) or microRNA-like RNAs in fungi (milRNAs) [1–3]. Once produced, sRNAs are loaded onto an RNA-induced silencing complex (RISC) to guide Argonaute ribonuclease (AGO) to identify complementary messenger RNAs (mRNAs) that will be silenced through cleavage, translation inhibition, or modification of chromatin [1, 2, 4]. Due to their contribution to PTGS, sRNAs play a versatile role in living organisms’ life cycles, including biotic interactions [5–8]. In addition, sRNAs can move bidirectionally and modulate communication between interacting organisms by regulating gene expression of recipient species through targeted gene silencing called cross-kingdom RNA silencing [7, 9–13]. Although the role of sRNAs in cross-kingdom RNA silencing between interacting organisms is established, including parasitic and mutualistic interactions, their role in beneficial interactions between fungal biocontrol agents (BCAs) and their plant hosts is yet to be explored thoroughly.

Fungal BCAs from the genera *Trichoderma* and *Clonostachys* can occupy diverse environmental niches and closely interact at inter-species and intra-species levels for nutrients and space. In addition to directly antagonizing fungal plant pathogens, some of these species can colonize plant roots and establish mutualistic associations with host plants by promoting health and priming induced immune response against pathogens [14–16]. For successful beneficial association, biocontrol fungi and host plants reprogram their genetic machinery and establish a molecular dialogue determining the degree of interactions [17–19]. RNA silencing has been shown to affect biocontrol fungi’s development, specialized metabolite production, and antagonistic activity [12, 20, 21]. Similarly, the role of fungal sRNA in fungus-plant interaction relevant for biocontrol is also considered. A recent study has shown that milRNAs of the biocontrol fungus *Trichoderma asperellum* can potentially target tomato genes involved in responses to ethylene and oxidative stress [22]. Similarly, three wheat miRNAs engaged in response to abiotic and biotic stress are shown to be downregulated during the interaction with *Trichoderma cremeum* and *Trichoderma atroviride* [23]. However, these findings fail to provide experimental evidence corroborating sRNA-mediated fungal-plant interaction and cross-kingdom RNA silencing. Furthermore, knowledge regarding how endogenous RNA silencing regulation could affect the relationship between biocontrol fungi and host plants is elusive. On the other hand, the role of plant sRNAs mediating the interaction between the biocontrol fungus *T. atroviride* and model plant *Arabidopsis thaliana* has recently been investigated [24]. In response to *T. atroviride* root colonization, *A. thaliana* showed induced expression of the RNA silencing machinery genes at local and systemic levels, which played a crucial role in the beneficial fungus-plant interactions by regulating expression pattern of the genes associated with plant growth and defense [24]. However, the precise plant sRNAs mediating the response to biocontrol fungi and their gene targets are unknown.

We aimed to investigate sRNA-mediated mechanisms regulating interactions between biocontrol fungi and plant hosts, studying both the role of endogenous RNA silencing and the potential for cross-kingdom RNA silencing. We used the filamentous fungus *Clonostachys rosea* and wheat plant as the fungal BCA and plant host to achieve the goal. *Clonostachys rosea* can colonize plant roots, including wheat, promoting plant health and inducing defense responses (beneficial fungus-plant interactions) against several fungal plant pathogens [14, 15, 25–28]. In addition, *C. rosea* can thrive as a necrotrophic mycoparasite and can antagonize plant pathogenic nematodes [26, 29–33]. To perform these functions, certain *C. rosea* strains are shown to regulate their genetic machinery to produce an arsenal of chemical compounds and proteins, including hydrolytic enzymes, small-secreted proteins, and transporters [18, 27, 32, 34–38]. The expression regulation of such compounds and proteins was recently shown to be partially mediated by sRNAs [21, 39]. Deleting *dcl2* resulted in mutants with altered expression of genes, including those involved in the production of hydrolytic enzymes, membrane transporters, specialized metabolites, and transcription factors [21]. Moreover, the resulting deletion mutant has been proven to have reduced growth in vitro, reduced antagonism towards *Botrytis cinerea*, and a reduced capacity to control *Fusarium* foot rot on wheat [21].

In the current study, we therefore aimed to investigate DCL-mediated gene expression regulations during *C. rosea*-wheat interactions. The objectives of the current work were (i) to identify *C. rosea* and wheat genes regulated during their interaction and (ii) to unravel the role of sRNAs in mediating gene expression regulation during *C. rosea*-wheat interaction to understand the fungus-plant interactions relevant to biocontrol. We hypothesized that the transcriptomic response in *C. rosea* towards wheat plants is DCL-dependent. We further hypothesized that alteration in RNA silencing pathways in *C. rosea* will influence the transcriptomic response of wheat plants towards *C. rosea*. We sequenced sRNAs and transcriptomes of *C. rosea* and wheat roots to verify these hypotheses during their interaction. In addition, we used previously generated *dcl1* and *dcl2* gene deletion mutants [21] to elucidate the role of sRNA on gene expression regulation at endogenous and cross-kingdom levels. This led us to identify plant and fungal candidate miRNAs, their potential gene targets, and genes with a potential role in beneficial fungus-plant interactions relevant for biocontrol.

## Results

### Deletion of dcl2 affects the root colonization ability of *C. rosea*

**Fig. 1:**
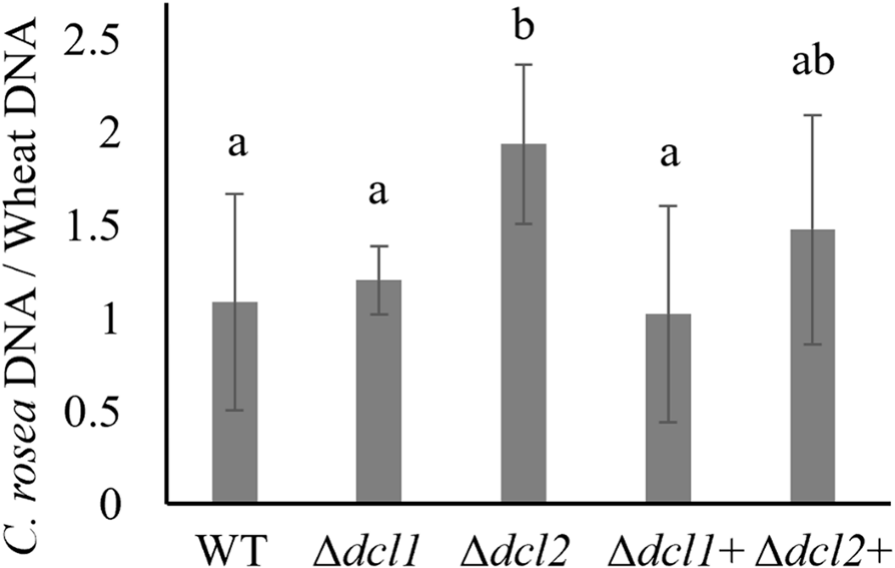
Determination of *C. rosea* root colonization in wheat roots. Wheat roots were harvested five days post-inoculation of *C. rosea* spores, and the fungal biomass was quantified using RT-qPCR. *C. rosea* colonization is expressed as the ratio between *C. rosea* DNA and wheat DNA. *Actin* and *Hor1* were used as target genes for DNA quantification for *C. rosea* and wheat, respectively. Error bars represent standard deviation based on five biological replicates. Different letters indicate statistically significant differences (*p* < 0.05) based on Fisher’s exact test

The role of DCL-mediated RNA silencing in beneficial fungus-plant interactions was investigated by comparing the root colonization ability of *C. rosea* wildtype (WT) and DCL deletion strains Δ*dcl1* and Δ*dcl2* (the *C. rosea* genome contains two genes *dcl1* and *dcl2* encoding for DCL proteins) and the complementation strains (Δ*dcl1* + and Δ*dcl2* +) generated in our previous study [16]. Root colonization ability was determined by measuring the biomass of *C*. *rosea* strains on wheat roots five dpi by quantifying the ratio between *C*. *rosea* DNA and wheat DNA with quantitative PCR (qPCR). Significantly (*p* < 0.019) higher *C*. *rosea*/wheat DNA ratios were detected on wheat roots inoculated with the Δ*dcl2* strain, compared with roots inoculated with the WT (Fig. 1). The result suggests an increased biomass of the Δ*dcl2* strain on wheat roots, compared with the WT. Complementation strain Δ*dcl2* + showed significant restoration of the phenotype. In contrast, no significant differences in *C*. *rosea*/wheat DNA ratio were found between WT and Δ*dcl1* inoculated wheat roots (Fig. 1).

### *C. rosea* and wheat RNA-seq during interactions

To investigate the sRNAs and mRNAs expression response of *C. rosea* strains and wheat during interactions, wheat roots (grown on moist filter plates in 9 cm diameter Petri plates) inoculated with *C. rosea* conidia were harvested at seven dpi, and sRNA and mRNA expressions were analyzed by RNA-seq. The mRNA sequencing produced between 33.51 and 26.03 million reads for each sample. Since the sequences contained read pairs from both the interacting species, the reads originating from *C. rosea* or wheat were identified by mapping to the respective genomes. As expected, in the samples coming from the interaction of *C. rosea* and wheat, the number of reads from *C. rosea* strains was significantly lower (ranging from 1.8 to 4 million reads) than the reads from wheat roots, amounting to 7% in the case of WT and 13% and 11% for the Δ*dcl1* and Δ*dcl2* mutants, respectively. Therefore, the possibility of missing out genes with low expression levels in *C. rosea* cannot be ruled out. A summary of the results from the mRNA sequencing is presented in Table 1 and Additional file 1: Table S1.

**Table 1:** Summary of transcriptome sequencing and number of reads assigned to wheat and *C. rosea*

Sample	*C. rosea* control	Wheat control	*C. rosea*-Wheat interactions		
			WT-Wr	Δ*dcl1*-Wr	Δ*dcl2*-Wr
Raw reads (in million bp)^b^	27.97	28.54	28.64	29.59	27.88
Clean reads (in million bp)^b^	27.83	28.29	28.35	29.36	27.73
Reads unique to *C. rosea*^a^	85.08	0.23	6.92	12.86	10.80
Reads assigned to *C. rosea* genes^a^	73.10	NA	5.74	10.95	9.37
Unassigned *C. rosea* reads^a^	11.12	NA	1.12	1.81	1.33
Reads not mapped to *C. rosea*^a^	0.85	NA	0.06	0.10	0.09
Reads unique to wheat^a^	0.55	85.44	76.10	70.36	74.02
Reads assigned to wheat genes^a^	NA	69.51	57.23	54.37	58.71
Unassigned wheat reads^a^	NA	13.39	11.54	10.01	10.32
Reads not mapped to wheat^a^	NA	2.54	7.32	5.98	5.00

^a^Percentages of clean reads. *WT*, *C. rosea* wildtype; *Wr*, wheat roots

^b^The average read number from three biological replicates

*WT*, *C. rosea* wildtype; *Wr*, wheat roots

The sRNA sequencing produced between 11.89 and 18.45 million reads per sample. After filtering out structural RNAs and reads shorter than 18 nt or longer than 32 nt, between 29 and 37% of reads were retained. Of these, between 21 and 30% of reads had an antisense match on the wheat genome, while between 2 and 6% of them, on average, mapped to the *C. rosea* genome. Conversely, up to 96% of these reads had a sense match on the wheat transcriptome, while this number never increased above 5% for *C. rosea* (Table 2, Additional file 1: Table S1).

**Table 2:** Summary of sRNA sequencing and number of reads assigned to wheat and *C. rosea*

Sample	*C. rosea* control	Wheat control	*C. rosea*-Wheat interactions		
			WT-Wr	Δ*dcl1*-Wr	Δ*dcl2*-Wr
Raw reads (in million bp)^b^	11.90	13.86	19.04	15.51	15.57
Clean reads (in million bp)^b^	9.65	12.21	16.28	13.37	13.41
Reads of 18–32 nt ^a^	39.85	33.45	30.14	32.45	35.77
Non-structural reads of 18–32 nt ^a^	36.96	30.37	27.39	28.88	32.39
Sense reads mapped to wheat transcriptome^c^	NA	81.68	89.74	91.09	95.95
Antisense reads mapped to wheat transcriptome^c^	NA	25.63	22.39	20.91	30.50
Sense reads mapped to CR transcriptome^c^	4.73	NA	2.83	2.92	4.40
Antisense reads mapped to CR transcriptome^c^	6.07	NA	1.83	1.93	1.91

^a^Percentages of clean reads. *WT*, *C. rosea* wildtype; *Wr*, wheat roots

^b^The average read number from three biological replicates

^c^Percentages of clean non-structural reads of 18–32 nt. *WT*, *C. rosea* wildtype; *Wr*, wheat roots

### The transcriptomic response of wheat during interaction with *C. rosea* involves genes associated with stress response and growth

**Fig. 2:**
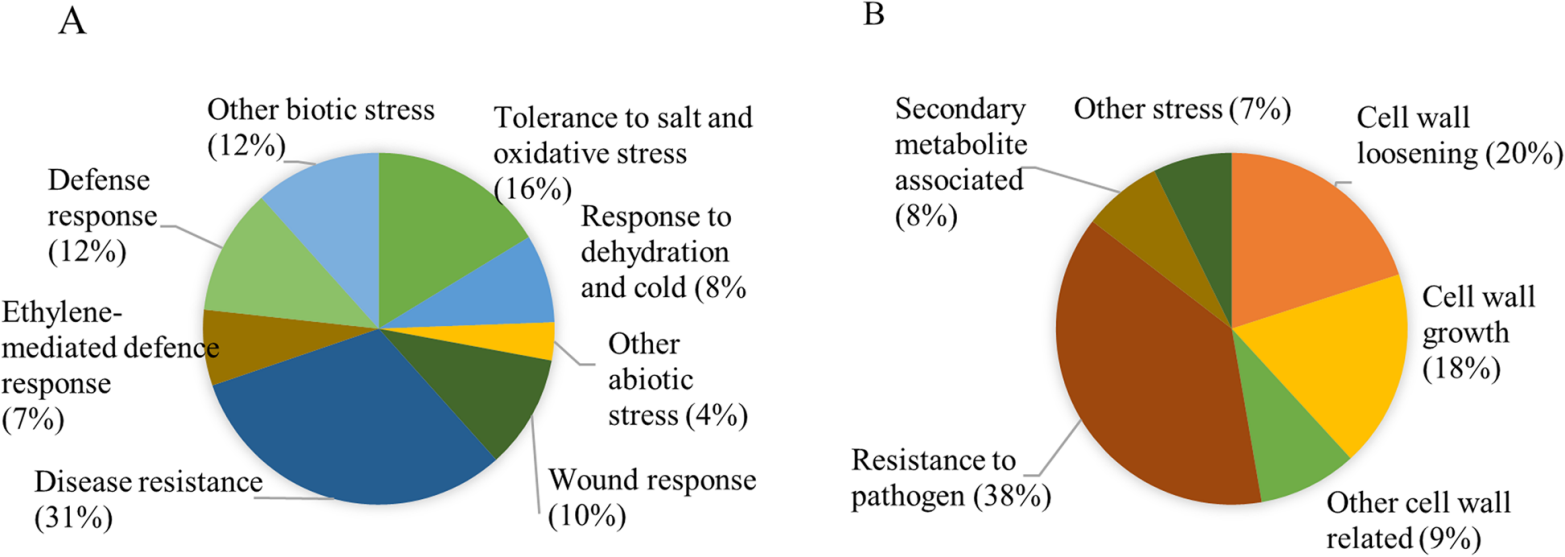
The transcriptomic response of wheat to interaction with *C. rosea*. **A** Pie chart showing the proportion of the stress-related genes in wheat upregulated during Cr-Wr. **B** Pie chart showing the proportion of stress-related genes, specialized metabolism, and cell wall-related genes downregulated during Cr-Wr

The expression profile of wheat transcripts during interactions with *C. rosea* WT (Cr-Wr) was compared to that of non-interaction wheat control to identify genes differentially expressed in wheat roots. In comparison to the control, 280 wheat genes were significantly upregulated (log2FC > 1.5), and 208 were downregulated (log2FC < −1.5) in wheat during Cr-Wr (Additional file 1: Table S2). Gene ontology analysis of upregulated genes is summarized in Additional file 2: Fig. S1 and Additional file 1:Table S3. Of the upregulated genes, 86 were identified as biotic, abiotic stress-related, and wound-responsive (Fig. 2A, Additional file 1: Table S4). Among these, fifty-three genes were associated with biotic stress tolerance. This includes leucine-rich repeat (LRR) and lectin protein kinases, nodulin-like proteins, disease resistance proteins, defensins, vicilin-like proteins, and ethylene-responsive transcription factors. At the same time, abiotic stress-responsive genes included late embryogenesis abundant (LEA) proteins (salt and oxidative stress tolerance) and dehydrins (dehydration and cold tolerance) (Fig. 2A, Additional file 1:Table S4). The top 20 most upregulated genes included a RGA5-like disease resistance protein; *Fusarium* resistance orphan protein Traescs4b01g106100.1; defensin-like 1 protein, which has antifungal activity [40]; vicilin-like seed storage proteins, known for inhibiting the spore germination and growth of filamentous fungi [41, 42]. In this group, we also identified a ubiquitinyl hydrolase 1 and an F-box protein, which affect several plant processes, including stress response [43], as well as a TAR1-like protein, putatively involved in auxin biosynthesis and consequently in hormone crosstalk and plant development [44, 45] (Additional file 1: Table S5).

During Cr-Wr, 208 wheat genes were downregulated (Additional file 1:Table S2). These genes were enriched in many biological processes but mainly related to the modification of plant cell walls and metabolic processes (Additional file 2: Fig. S1, Additional file 1: Table S3). Among the downregulated genes, 55 were associated with resistance to pathogens and cellular growth by performing cell wall loosening or modification (Fig. 2B, Additional file 1: Table S4). Moreover, 26 downregulated genes encoded for expansin-like proteins and xyloglucan endotransglucosylase/hydrolases, polygalacturonases, a cellulose synthase-like protein D1 and a dirigent protein 5-like proteins predicted to be involved in cell wall loosening and modification (Fig. 2B, Additional file 1: Table S4). The top 20 significantly downregulated genes included a nucleotide binding site (NBS)-LRR resistance protein mediating pathogen sensing and host defense [46, 47]; a pathogenesis-related protein 1 with antifungal activity [48]; a peroxidase 5-like involved in ROS burst; a zealexin A1 synthase-like involved in the synthesis of protective phytoalexins [49]; two chalcone synthases, which affect resistance by influencing the salicylic acid response and the accumulation of flavonoid phytoalexins [50, 51]; and a serpin, a class of protease inhibitors that can have a role in the inhibition of plant-hypersensitive responses [52] (Additional file 1: Table S5). These results suggest that the root colonization by *C. rosea* resulted in transcriptional reprograming of wheat genes associated with stress response and growth, indicating a plausible trade-off between defense and development.

### Clonostachys rosea interactions with wheat roots triggered transcriptional reprograming of fungal genes encoding for CAZymes, membrane transporters and effectors

In *C*. *rosea*, 1908 genes were upregulated during Cr-Wr, compared to *C*. *rosea* control, while 1262 were downregulated (Additional file 1: Table S2). The biological processes in the genes upregulated during Cr-Wr interactions mostly referred to an increase in the catabolism in many types of carbohydrates (Additional file 2: Fig. S1, Additional file 1: Table S3). This can be attributed to the fact that 229 upregulated genes were encoded for putative CAZymes, and most of them (163) were reported as secreted in a previous study [53]. Among the CAZymes, 134 were glycoside hydrolases (GHs), and 87 of them had carbohydrate-binding modules (CBMs), the most frequent of which was CBM1, present in 55 proteins (Additional file 1: Table S6). Even if GHs were the most common type of CAZyme, the most numerous single class was auxiliary activities (AA) family 9 (21 genes), involved in the degradation of cellulose. The transmembrane transport, in particular, can be related to the upregulation of 135 major facilitator superfamily (MFS) and 13 ATP-binding cassette (ABC) transporters, classes that have been proven essential in the antagonistic activity of *Clonostachys* species [32]. In particular, the most numerous classes of upregulated MFS transporters were 2.A.1.1 (sugar porters), 2.A.1.14 (organic cation transporters) and 2.A.1.2 (drug: H^+^ antiporter), with 45, 31, and 28 members respectively. In addition, we identified 34 effector genes upregulated during Cr-Wr compared to the control (Additional file 1: Table S6). The 20 *C. rosea* genes most upregulated during Cr-Wr encoded nine proteins with putative roles in the polysaccharide (cellulose, hemicellulose, xylan, and lignin) catabolism. These enzymes are also associated with the degradation of the plant cell walls [54–56]. Moreover, 13 of these 20 genes encode for putative secreted effectors, suggesting their role in affecting the plant immune response (Additional file 1: Table S7). The 20 *C. rosea* genes most downregulated during the interaction with wheat included three transcription factors, a secreted putative effector, a non-ribosomal peptide synthetase (NRPS) involved in the synthesis of an unknown specialized metabolite, a superoxide dismutase participating in defense from reactive oxygen species (ROS) and four membrane transporters (Additional file 1: Table S7). Summarizing, the data highlights how the interaction with wheat roots triggers the transcriptional reprogramming of *C. rosea* genes associated with transport and carbohydrate catabolism.

### Deletion of *C. rosea* dcl1 and dcl2 altered the transcriptomic response of wheat roots during the interaction

**Fig. 3:**
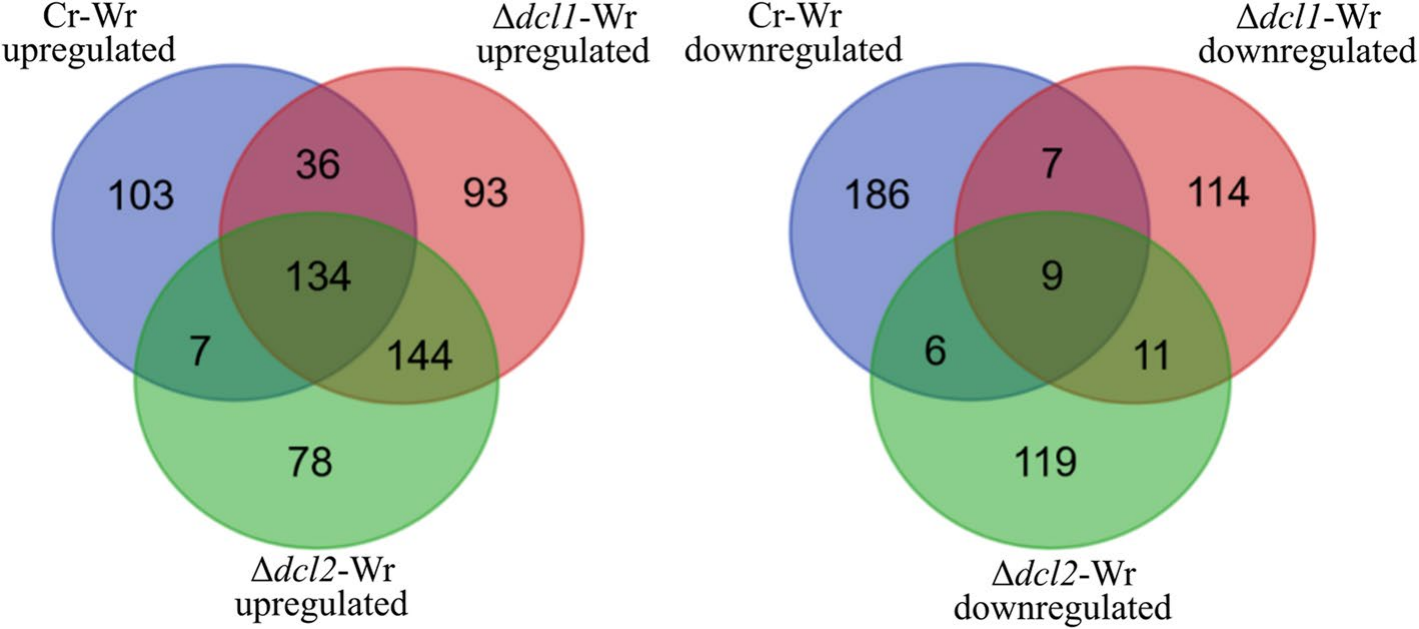
The number of differentially expressed wheat genes during the interactions with *C. rosea* WT or DCL gene deletion strains. The Venn diagram was generated with https://bioinformatics.psb.ugent.be/webtools/Venn/

To investigate whether deletion of *dcl1* and *dcl2* in *C. rosea* can affect the gene expression regulation in wheat roots during Cr-Wr, the gene expression pattern of wheat roots during the interaction with DCL1 deletion strain (Δ*dcl1*-Wr) and DCL2 deletion strain (Δ*dcl2*-Wr) was analyzed and compared to Cr-Wr and wheat control. We identified 144 wheat genes upregulated during the interaction with the DCL deletion mutants compared to wheat control but not during Cr-Wr. On the contrary, 93 and 78 genes were upregulated only during Δ*dcl1*-Wr and Δ*dcl2*-Wr, respectively (Fig. 3). Only eleven genes were downregulated during the response to both mutants but not to the WT, while 114 and 119 were uniquely downregulated during Δ*dcl1*-Wr and Δ*dcl2*-Wr*,* respectively. The differentially expressed wheat genes could be divided into nine co-expression modules, which showed how the deletion of *C. rosea dcl* genes affected the transcriptomic response of wheat. In particular, the module eigengenes (ME) of modules one, two, and eight showed a significant correlation with one of the deletion mutants, while they were not correlated with Cr-Wr. Vice versa, ME_4, ME_7, and ME_9 were negatively correlated with the deletion mutants while being either non-correlated or positively correlated with Cr-Wr (Additional file 2: Fig. S2). In summary, our results showed a shift in the transcriptomic response of wheat roots during Δ*dcl1*-Wr and Δ*dcl2*-Wr compared to Cr-Wr, suggesting a role of *C. rosea* sRNAs in regulating plant-fungal interactions.

### Interaction with dcl deletion strains affects the expression pattern of wheat genes associated with stress response, metabolism and growth

**Fig. 4:**
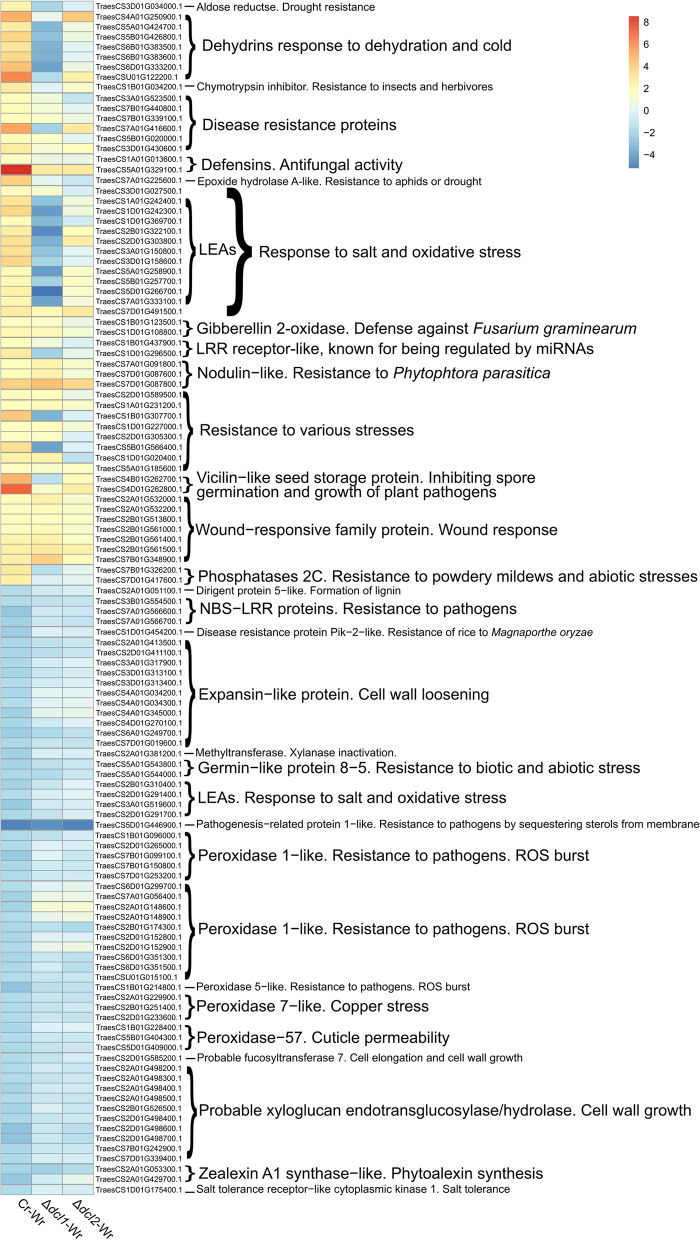
The heatmap shows the expression (Log2FC) of selected wheat genes of interest. All genes were differentially expressed during Cr-Wr (log2(FC) > 1.5 or < −1.5 and FDR < 0.05) but not during both or either Δ*dcl1*-Wr or Δ*dcl2*-Wr. Wr indicates wheat roots; Cr indicates *C. rosea *wildtype

**Fig. 5:**
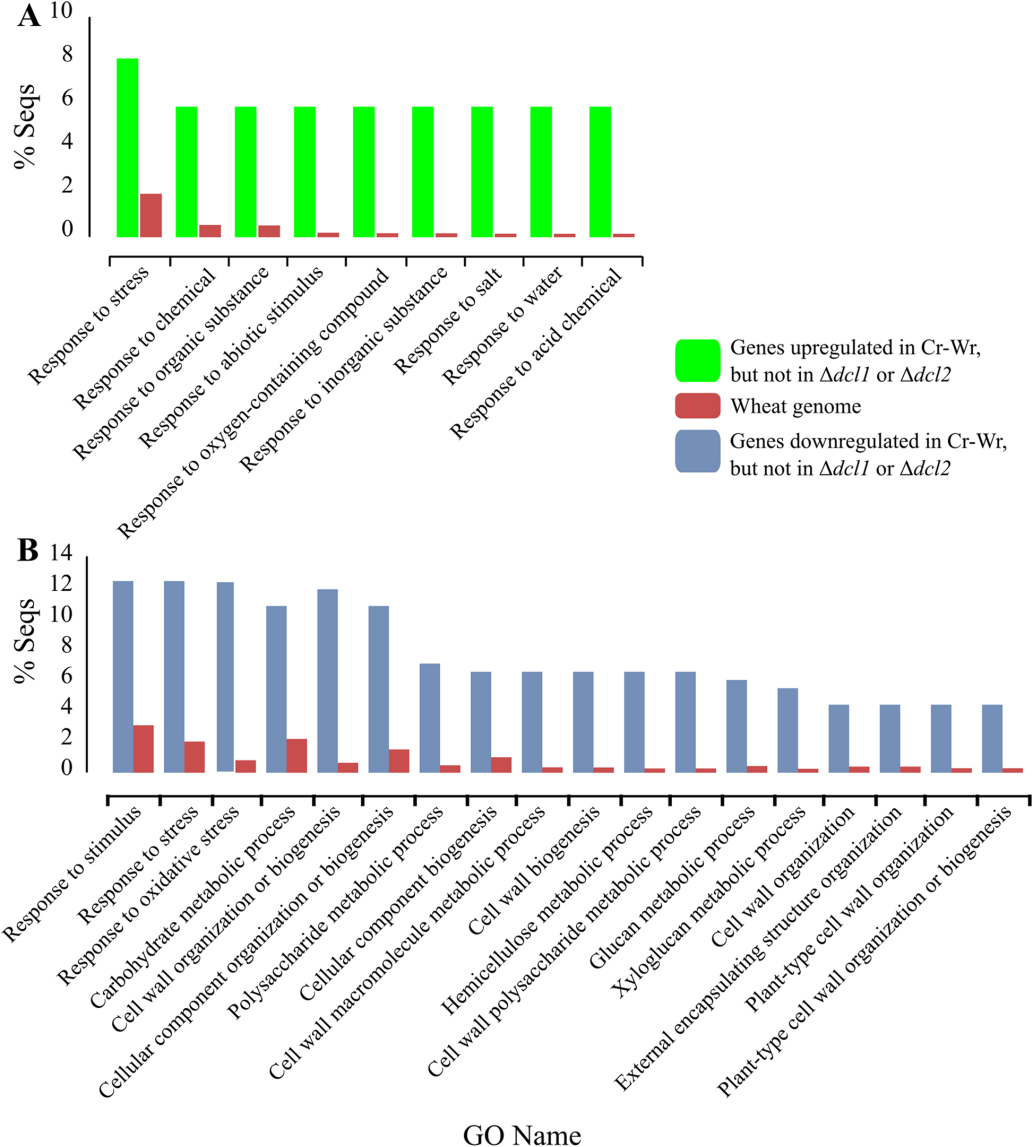
Percentage of genes annotated with gene ontology terms referring to biological processes enriched in wheat genes upregulated (**A**) or downregulated (**B**) during the interaction between wheat and the WT but not when the plant interacted with the Δ*dcl1* or Δ*dcl2* mutant. For each of these GO terms, the percentage of genes having the term in the whole wheat genome is compared with the percentage of genes with the term in the situation of enrichment (FDR < 0.05)

Among 146 wheat genes that were upregulated during Cr-Wr but not during either Δ*dcl1*-Wr or Δ*dcl2*-Wr, 65 were associated with stress response (Fig. 4, Additional file 1: Table S6). The GO term analysis showed that all terms enriched in these genes were related to the response to several stress-related factors (Fig. 5A). More specifically, this group included two protein phosphatases 2C, interacting with ADP-ribosylation factors involved in resistance to powdery mildews and abiotic stresses [57] and eleven LEA proteins necessary for tolerance of salt and oxidative stress [58]. Both protein classes are regulated by abscisic acid, and the same is true for membrane proteins PM19L-like, one of which had DCL-dependent expression in the current study [59–61].

Many other genes, upregulated in Cr-Wr but not in Δ*dcl1*-Wr or Δ*dcl2*-Wr, were related to resistance to various abiotic stresses. We could detect seven DHN dehydrins involved in response to dehydration and cold, one H-type thioredoxin mediating responses to oxidative stresses [62, 63] and one aldose reductase, whose overexpression improves drought resistance in transgenic plants [64]. Other detected genes were involved in resistance to pathogens, such as one Bowman-Birk type trypsin inhibitor-like isoform X2, which can inhibit in vitro growth of *F. graminearum*, *Fusarium culmorum*, and *Fusarium tritici* [65], and one premnaspirodiene oxygenase-like protein involved in resistance to *Phytophthora capsici* in black pepper [66]. Two defensin proteins also fall in this group, and similar proteins have an antifungal activity carried out through cell wall permeabilization [40]. We also identified two LRR receptor-like serine/threonine-protein kinases, a class involved in resistance to *Puccinia triticina* and *Plasmopara viticola*, and also known for being regulated by miRNAs [67–69]. Other DCL-dependent genes seem to be involved in resistance to both biotic and abiotic stresses, such as an epoxide hydrolase A-like, similar to genes involved in resistance to aphids and others targeted by drought-responsive miRNAs [70, 71], or one NAC protein, a class involved in drought resistance, sensitivity to abscisic acid (ABA), lignin biosynthesis, and resistance to *F. graminearum* and *Puccinia triticina* [72–74] (Fig. 4: Additional file 1: Table S6). In summary, this data suggests that while the interaction with *C. rosea* WT causes the upregulation of many stress-responsive genes in the plant, many of these genes are not similarly affected during the interaction with *C. rosea dcl* deletion mutants.

Deletion of DCL genes, moreover, caused the lack of downregulation of 199 wheat genes during Δ*dcl1*-Wr or Δ*dcl2*-Wr, which were downregulated during Cr-Wr (Additional file 1: Table S2, Fig. 3). These genes were enriched mainly in biological processes related to the synthesis, organization, and modification of the cell wall (Fig. 5B, Additional file 1: Table S3). Eleven of these genes were encoding for expansin-like proteins with a role in plant cell wall loosening, while three others were peroxidases-57, whose overexpression increases cuticle permeability in *A. thaliana* [75]. These include a methyltransferase involved in xylanase inactivation during *F. graminearum* infection [76], three resistance proteins of class NBS-LRR, 14 peroxidases of classes 1, 2, and 5 involved in resistance to biotic stress [77–79], and a disease resistance protein Pik-2-like, involved in resistance of rice to *Magnaporthe oryzae* and upregulated in wheat during *Blumeria graminis* infection [80, 81] (Fig. 4; Additional file 1: Table S6). In summary, this data suggests that, while during Cr-Wr, the plant downregulates many genes associated with metabolic processes, growth, and stress response, many of these genes are not similarly affected during the interaction with the *C. rosea dcl* deletion mutants.

### DCLs-mediated gene expression regulation in *C. rosea* during interaction with wheat roots

**Fig. 6:**
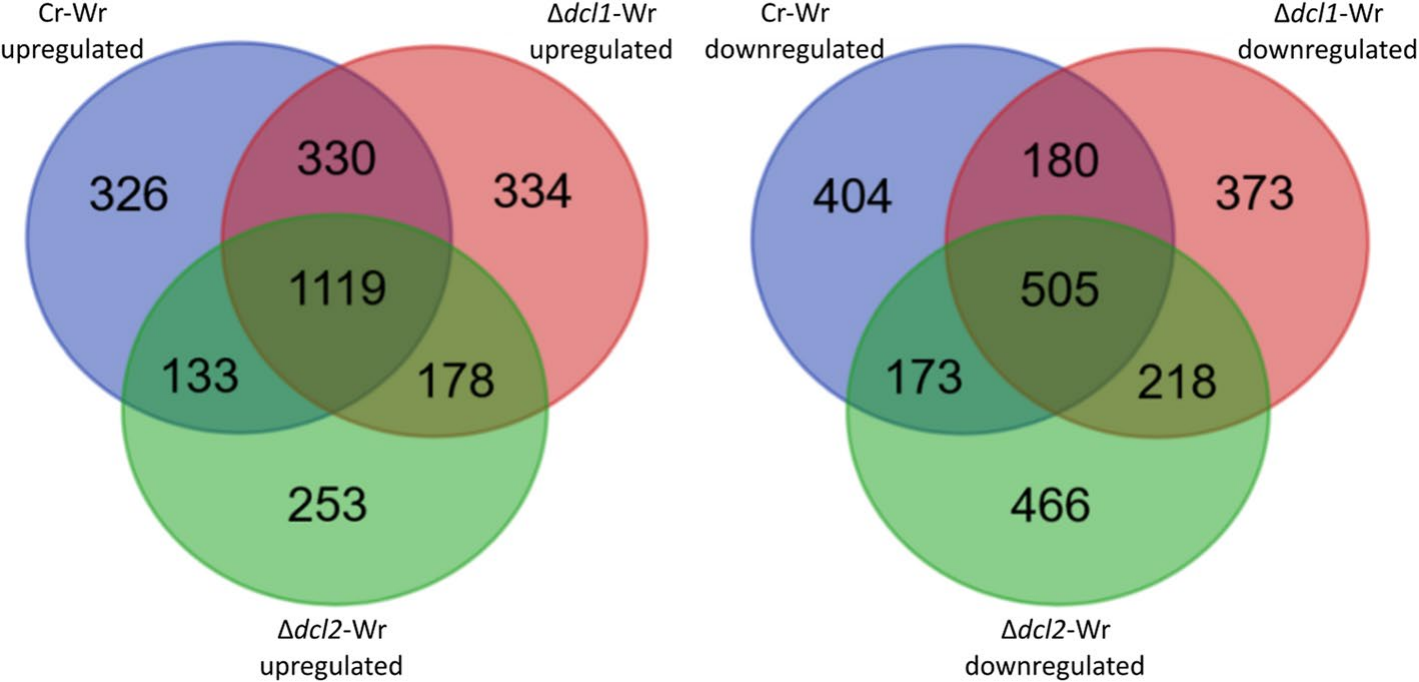
Number of differentially expressed *C. rosea* genes during the interaction with wheat roots. Venn diagram generated with https://bioinformatics.psb.ugent.be/webtools/Venn/

The deletion of the *dcls* affected the expression of multiple *C. rosea* genes during the interaction with wheat roots. Five hundred and twelve genes were upregulated in the Δ*dcl1* mutant but not in the WT, while this number was 431 for the Δ*dcl2* mutant. The number of downregulated genes in the *C. rosea* mutants but not in the WT corresponded to 591 genes in Δ*dcl1* and 684 in Δ*dcl2* (Fig. 6). The differentially expressed *C. rosea* genes were divided into nine co-expression modules, each showing a significant difference in expression between Cr-Wr and the deletion mutants, further underlining the importance of DCL-dependent RNA silencing in regulating gene expression during the interaction with wheat (Additional file 2: Fig. S3).

Similarly, the expression of 789 *C. rosea* genes, which were upregulated during Cr-Wr, were not upregulated during Δ*dcl1*-Wr or Δ*dcl2*-Wr, indicating DCL-mediated gene expression regulation. Conversely, 757 genes were downregulated during Cr-Wr but not during Δ*dcl1*-Wr or Δ*dcl2*-Wr.

Since many *C. rosea* genes encoding for putative CAZymes and effectors were upregulated during Cr-Wr, we analyzed if their expression was DCL-mediated. Fifty-four CAZymes genes, all upregulated in Cr-Wr, were not upregulated in *C. rosea dcl* deletion strains during the interaction with wheat roots, 32 of which were predicted to be secreted in a previous work [53] (Additional file 1: Table S6). Thirty-four putative effector encoding genes were upregulated in Cr-Wr but not in the *C. rosea dcl* deletion strains (Additional file 1: Table S6).

### Identification of wheat miRNAs responsive to *C. rosea* interactions and their putative endogenous and cross-kingdom gene targets

**Fig. 7:**
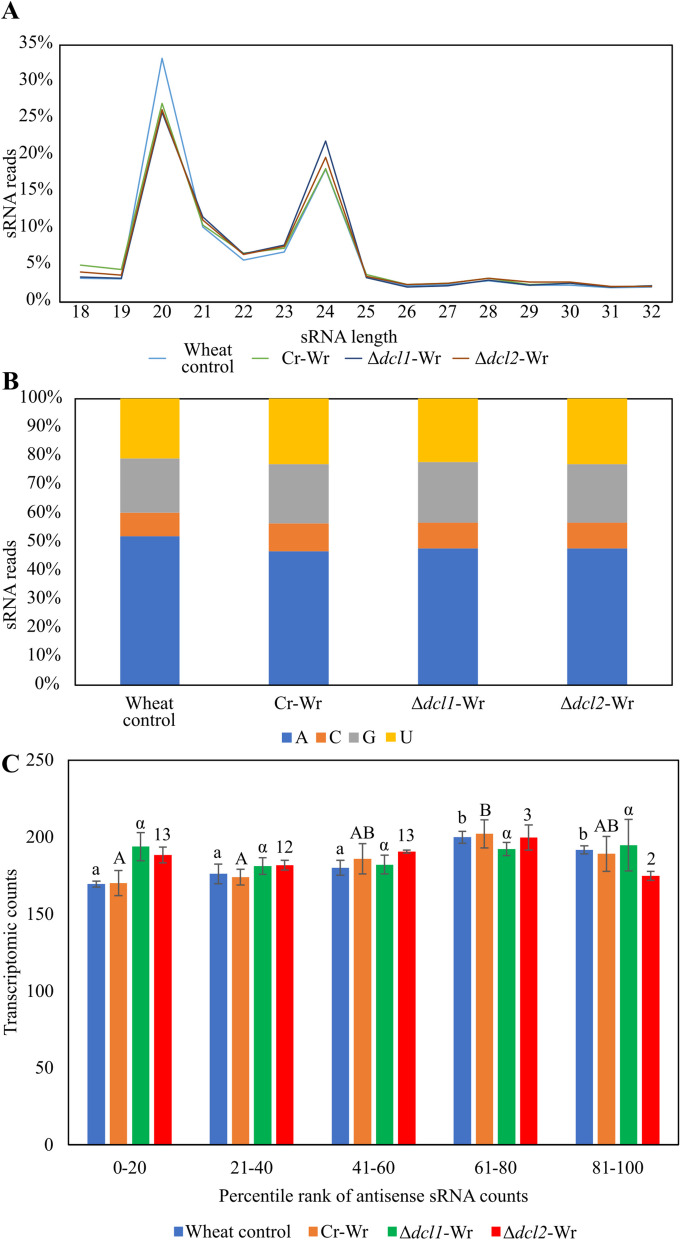
**A** Read length distribution of sRNA reads mapped to wheat. **B** 5′ base distribution of sRNA reads mapped to wheat. **C** Average transcriptome read counts of wheat genes, depending on their percentile rank of antisense sRNA counts. Percentile ranks were assigned to each gene based on its antisense sRNA counts. Therefore, genes in the group “1–20” are the 20% of genes with the lowest amount of antisense sRNAs mapping to them, while genes in the group “81–100” are the ones with the highest number of antisense sRNAs. Genes with an antisense sRNA count of zero were not considered. Lowercase, uppercase, Greek letters and numbers indicate groups not significantly different according to separate Tukey tests with a maximum *p*-value of 0.05

To provide insights into gene expression regulation during Cr-Wr, we investigated sRNA-mediated wheat gene expression regulation by analyzing sRNA characteristics and their expression patterns in response to *C. rosea* root colonization. The length distribution of sRNA reads showed a higher proportion of reads with 20 nt (34%) and 24 nt of length (Fig. 7A). The 5′ terminal nucleotide composition analysis showed a higher proportion (47–52%) of the reads with 5′ end adenine (5′—A). At the same time, both guanine and uracil were present at 5′ of 20% of the reads, and cytosine was the lowest base in that position, covering less than 10% of the reads (Fig. 7B). We compared the characteristics of wheat sRNAs produced in the control treatment to those produced during Cr-Wr. The analysis showed a reduction from 33 to 27% in sRNAs with a size of 20 nt in wheat during Cr-Wr compared to control wheat roots, while no difference was found between Δ*dcl1*-Wr or Δ*dcl2*-Wr and Cr-Wr (Fig. 7A). The correlation between mRNA mapping and antisense sRNA mapping was analyzed on every transcript to identify their putative gene targets. On average, transcripts with high numbers of antisense sRNAs mapping to them were not less expressed (lower transcriptome counts) than those with few antisense sRNAs mapping to their location (Fig. 7C).

Seven novel and 649 known miRNAs were detected in wheat samples in at least 50 copies (Additional file 1: Table S8), ranging from 19 to 23 nt in length. In contrast to the size distribution of total sRNAs, a higher number (47%) of miRNAs were 21 nt in length (Additional file 2: Fig. S4A). Among these, six miRNAs were downregulated during Cr-Wr compared to wheat control, while three were upregulated. The deletion of *dcl1* in *C. rosea* impacts the miRNA-based response of wheat, with seven miRNAs upregulated and four downregulated during Δ*dcl1*-Wr compared to Cr-Wr interaction (Table 3). In the case of the *dcl2* deletion*,* we detected only three upregulated miRNAs and one downregulated when comparing Δ*dcl2*-Wr with Cr-Wr (Table 3, Additional file 2: Fig. S4B).

**Table 3:** Sequence, length, and expression level of differentially expressed wheat miRNAs and *C. rosea* milRNAs identified during interactions. Values in bold indicate differential expression with FDR < 0.05

miRNAs/milRNA	Total read count	Mature sequence	Length	Log2FC expression		
				Cr-Wr VS Control	Δ*dcl1*-Wr VS Cr-Wr	Δ*dcl2*-Wr VS Cr-Wr
**Differentially expressed *C. rosea* milRNAs**						
cro-mir-1	68,544	UAGAAUUCGGGGUAGAAU	18	** − 1.83**	-0.07	** − 5.84**
cro-mir-2	2032	UAGAAUUCGGGGUAGAAUG	19	** − 2.73**	0.49	** − 4.35**
cro-mir-3	33,147	UUAGCCUCGAGACUUUGCA	19	** − 3.74**	0.27	** − 4.49**
cro-mir-4	1994	UCAGCCUCGAGACUUUGCC	19	** − 2.92**	0.77	** − 6.01**
cro-mir-10	875	UCGGUGGGAUGUUUGAGACU	20	** − 4.74**	1.11	** − 3.09**
cro-mir-11	955	UAGAGUUUUUGGAGAUGCU	19	** − 2.42**	0.49	** − 6.38**
cro-mir-36	32,160	UCAAACACAAUUAGCGGUC	19	** − 1.9**	0.78	** − 6.02**
cro-mir-73^a^	48,980	UCUGAAGGUCGUGUGUUC	18	**5.65**	** − 2.69**	** − 2.74**
cro-mir-77^a^	683	UAUGCCUAGGCUUGUGCGA	19	** − 3.54**	− 0.93	** − 4.9**
cro-mir-5	94	UUGCAAUGAUUUGCAUUUCGC	21	** − 3.76**	− 2.17	− 0.88
cro-mir-6	1875	UAGGACUCGAGUAGUUAUAAC	21	** − 5**	0.94	− 2.47
cro-mir-9	447	UCGGACGUAUAUUGACUACUC	21	** − 3.79**	− 0.95	− 4.05
cro-mir-13	11,099	UUCUUCCUUGAUGCGUCCC	19	** − 4.62**	0.11	− 3.85
cro-mir-30	187	UGCCUGUCUGAGCGUCAUU	19	** − 2.9**	2.15	1.12
cro-mir-74^a^	460	CACGAUGUCCCGUAUCCGACGU	22	** − 2.78**	− 0.73	− 3.21
cro-mir-76^a^	700	UGUUUCUUUGUUUUUGCCU	19	** − 4.14**	0.97	− 2.46
**Differentially expressed Wheat miRNAs**						
mir_12061_x13	122	UGUAGAUACUCCCUAAGGCUU	21	** − 1.98**	**1.71**	0.01
mir_18750_x1	112	UGUAGAUACUCUCUAAGGCUU	21	** − 2.53**	**2.22**	0.48
mir_16010_x2	114	UGUAGAUACUCCCUAAGGCU	20	** − 2.32**	**2.01**	0.27
mir_17532_x1	118	UGUAGAUACUCCCUAGGGCUU	21	** − 2.23**	**1.96**	0.15
mir_19460_x1	125	CACCAACCGGUACUAAUGGGCAUC	24	** − 2.01**	**2.1**	1.55
mir_13110_x8	172	UCGGACCAGGCUUCAUUCCUU	21	** − 1.51**	1.48	0.68
mir_18139_x1	179	CGCCCCACGGUGGGCGCCA	19	**1.65**	− 0.8	− 0.74
mir_16988_x1	296	GUGGAUGAUGAGAUCACAAGUAA	23	**1.75**	− 0.52	− 0.95
mir_15432_x2	140	GCCCCACGGUGGGCGCCA	18	**2.34**	− 0.74	− 1.1
mir_13653_x6	10,902	GCCCGUCUAGCUCAGUUGGU	20	0.25	** − 1.68**	** − 1.5**
mir_16507_x1	83,527	CCGACCUUAGCUCAGUUGGU	20	0.32	** − 1.51**	− 1.31
mir_18684_x1	201	GACCUGUAUGGGGCACCA	18	0.69	** − 1.9**	− 0.93
mir_19043_x1	3970	AACCUUGUGGUCGUGGGUUC	20	0.95	** − 1.76**	− 1.39
mir_15848_x2	30,986	CCCGCCUUGCACCAAGUGAAU	21	− 0.36	**1.54**	0.83
mir_16416_x1	153	CAUCUCUCCUGUAGAAAUAGGCAC	24	− 0.8	**1.56**	0.9
mir_17663_x1	8712	UUUCCCGGCUAGUGCACC	18	− 0.51	− 0.44	**1.58**
mir_16687_x1	1322	AACUACAAUCUGAGGCUU	18	− 0.71	0.11	**2.42**
mir_18451_x1	689	CCACAGGCUUUCUUGAACUG	20	− 0.43	0.12	**2.51**

^a^Novel *C. rosea* milRNAs. *WT*, *Cr*,* C. rosea* wildtype; *Wr*, wheat roots

After target prediction with multiple tools and removal of targets not supported by opposite expression (that is, to consider a transcript as putatively targeted by a miRNA, it had to be upregulated when the miRNA was downregulated), 24 putative endogenous gene targets were identified for seven differentially expressed wheat miRNAs (Additional file 2: Fig. S4C; Additional file 1: Table S9). However, only four of the targets showed a significant Spearman correlation of less than − 0.7 between target mRNA and targeting miRNA counts (Table 4).

**Table 4:** Putative *C. rosea* milRNAs and wheat miRNAs and their putative endogenous and cross-kingdom gene targets of interest. At least two target prediction tools have predicted all putative targets. The plant-based tools psRNATarget, Targetfinder, psROBOT, and TAPIR were used for all putative miRNA-target couples. Moreover, animal-based tools of PITA, Miranda, TargetSpy, and simple seed analysis were utilized through the sRNA toolbox for self-targets of *C. rosea* milRNAs. All the shown targets also show opposite expressions to the targeting milRNAs (a target needs to be upregulated when the targeting milRNA is downregulated), with Spearman correlation, which is significant, <  − 0.4, and also significantly lesser than the correlation to the average transcript in the same organism. Log2Fc values are in bold when significant (adjusted *p*-value < 0.05)

miRNAs/milRNA	Target transcript	Spearman correlation	Transcript expression Log2Fc			Target gene family	Putative function
			Cr-Wr	Δ*dcl1-Wr*	Δ*dcl2-Wr*		
**Endogenous targets, *C. rosea***							
cro-mir-36	CRV2T00011673_1	− 0.94	**1.41**	**1.07**	**1.59**	ABC transporter	Multidrug resistance
cro-mir-76	CRV2T00005739_1	− 0.95	**3.11**	**2.91**	**4.12**	Acid phosphatase	Salt stress resistance
cro-mir-76	CRV2T00021953_1	− 0.96	**3.11**	**2.97**	**4.13**	Acid phosphatase	Salt stress resistance
cro-mir-3	CRV2T00011242_1	− 0.82	**0.31**	**0.52**	**1.14**	Cytochrome P450 monooxygenase	Xenobiotic detoxification and others
cro-mir-1 cro-mir-2	CRV2T00016330_1	− 0.9 − 0.87	**0.5**	**0.48**	**0.81**	MFS transporter	Allantoate permease
cro-mir-30	CRV2T00004939_1	− 0.77	**2.71**	**1.98**	**1.78**	MFS transporter 2.A.1.2	Multidrug resistance
cro-mir-76	CRV2T00009699_1	− 0.83	**0.47**	0.26	**1.09**	MFS transporter 2.A.1.3	Multidrug resistance
cro-mir-5	CRV2T00017422_1	− 0.8	**9.73**	**9.1**	**8.22**	Probable apoplastic effector	Unknown
cro-mir-36	CRV2T00000889_1	− 0.83	**2.03**	**1.74**	**1.98**	Transcription factor	Glucose-induced endocytosis and carbon catabolite derepression
cro-mir-3	CRV2T00017200_1	− 0.91	**1.07**	**0.54**	**2.16**	Transcription factor	Gene expression regulation
cro-mir-5	CRV2T00000691_1	− 0.78	**0.49**	**0.7**	**0.44**	Transcription factor	Gene expression regulation
**Endogenous targets, wheat**							
mir_15432_x2	TraesCS1D01G107100.1	− 0.77	− **1.17**	− 0.52	− 0.20	Mixed-linked glucan synthase 8	Cellulose biosynthesis
mir_15848_x2	TraesCS3D01G258300.1	− 0.83	0.32	− 0.56	− 0.37	ABC transporter	Unknown
mir_16507_x1	TraesCS4D01G102700.1	− 0.82	− 0.21	0.38	0.42	L-type lectin receptor kinases	Sensing pathogens and activating defense responses
mir_15432_x2	TraesCS2B01G311600.1	− 0.77	− **0.58**	− 0.4	− 0.48	transport protein Sec24B-like	Vesicle trafficking
mir_18139_x1		− 0.76					
***C. rosea* transcripts targeted by wheat miRNAs**							
mir_17532_x1	CRV2T00016916_1	− 0.82	**5.63**	**4.00**	**4.51**	PKS	Synthesis of an antimicrobial compound
mir_16010_x2		− 0.85					
mir_12061_x13		− 0.83					

Cross-kingdom target prediction identified six potential cross-kingdom gene targets in *C. rosea* for five wheat miRNAs, which showed an inverse relation in the expression between miRNAs and their corresponding gene targets (Additional file 1: Table S9). However, only one gene (CRV2T00016916_1) showed a significant negative correlation (Spearman correlation ≤  − 0.82) and was identified as a gene target for three wheat miRNAs mir_17532_x1, mir_16010_x2, and mir_12061_x13 (Table 4). This gene was identified as polyketide synthase gene *pks29,* shown to be involved in the synthesis of an antifungal polyketide [35], and it was strongly upregulated during the interaction between wheat and *C. rosea*.

### Identification of *C. rosea* miRNAs differently expressed during the interaction with wheat and their potential endogenous and cross-kingdom gene targets

**Fig. 8:**
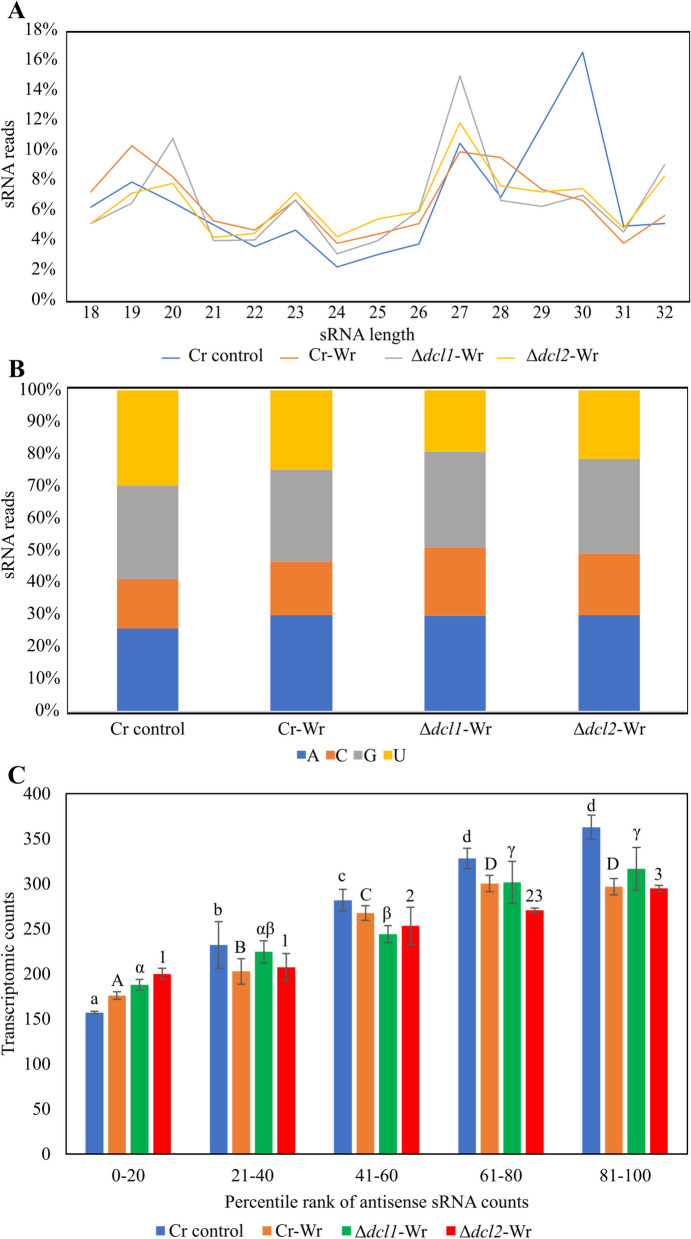
**A** Read length distribution of sRNA reads mapped to *C. rosea*. **B** 5′ base distribution of sRNA reads mapped to *C. rosea*. **C** Average transcriptome read counts of *C. rosea* genes, depending on their percentile rank of antisense sRNA counts. Percentile ranks were assigned to each gene based on its antisense sRNA counts. Therefore, genes in the group “1–20” are the 20% of genes with the lowest amount of antisense sRNAs mapping to them, while genes in the group “81–100” are the ones with the highest number of antisense sRNAs. Genes with an antisense sRNA count of zero were not considered. Lowercase, uppercase, Greek letters and numbers indicate groups not significantly different according to separate Tukey tests with a maximum *p*-value of 0.05

The sRNA characteristic and expression pattern were analyzed to investigate sRNA-mediated gene expression regulation in *C. rosea* during interaction with wheat roots. The analysis of read length distribution showed peaks of *C. rosea* sRNAs with a size of 19 nt (7–10%), 23 nt (5–7%), and 27 nt (10–15%). Moreover, *C. rosea* control had a higher proportion of 30 nt (17%) sRNAs than Cr-Wr. A peak in sRNAs with a size of 20 nt (11%) was recorded in the Δ*dcl1* strains compared to the other situations (Fig. 8A). The analysis of 5′ terminal nucleotide composition showed a higher proportion of 5′ – end uracil (5′ – U) and 5′ end guanine (5′ – G) in *C. rosea* control, both occupying around 30% of the reads, followed by 5′ adenine (5′ – A, 25%) and 5′ cytosine (5′ – C, 15%). However, during the interactions, the 5′ – A proportion increased to 30%, while the 5′ – U decreased to 25%. The 5′ base distribution was also affected by the deletion of the *dcl* genes, with a reduced proportion of sRNA reads with 5′ – U (20%) and an increased proportion of 5′ C (20%) during Δ*dcl*-Wr (Fig. 8B). The gene targets of these sRNAs were predicted by mapping to the transcripts. The number of antisense sRNA reads mapped to a *C. rosea* transcript did not correspond on average with a reduced expression (Fig. 8C).

Our analysis identified 16 known and five novel milRNAs (Additional file 1: Table S8), almost half of which were 19 nt in length (Additional file 2: Fig. S5A). Of these, 15 milRNAs were downregulated during Cr*-*Wr interaction, compared to *C. rosea* control. Nine milRNAs were downregulated during Δ*dcl2*-Wr compared to Cr-Wr, suggesting their origin was DCL-dependent, while one milRNA was downregulated during Δ*dcl1*-Wr (Table 3, Additional file 2: Fig. S5B). Only five of the Dicer-dependent milRNAs were absent (< 5 reads) from the mutants (cro-mir-2, cro-mir-4, cro-mir-10, cro-mir-11 and cro-mir-77), while the others showed strong downregulation. Differentially expressed *C. rosea*’s milRNAs had 480 putative endogenous targets. Three hundred and twenty showed a significant inverse correlation between gene target expression and their corresponding milRNAs (Spearman correlation >  − 0.4), and in 31 cases, a Wilcoxon rank sum test determined that the anticorrelation was significantly higher with the target than with the average transcript (Additional file 1: Table S9). These genes included one ABC transporter, three MFS transporters, and three transcription factors, as well as two acid phosphatases, one Cytochrome P450 monooxygenase, and one putative apoplastic effector (Table 4).

Many endogenous gene targets (405) were predicted to be targeted by downregulated *C. rosea* milRNAs. These consisted of genes encoding for transcription factors (eleven genes), putative effectors (twelve genes), MFS transporters (nineteen genes), core enzymes of specialized metabolite gene clusters (two genes), RNA helicases (two genes), and chromatin remodeling proteins (two genes) (Additional file 1: Table S9). On the contrary, only one milRNA (cro-mir-73) was upregulated during Cr-Wr, compared to the control, and it was predicted to target three genes: a transcription factor, a proteolytic enzyme and a protein involved in guanosine tetraphosphate metabolism (Additional file 1: Table S9).

The nine DCL2-dependent milRNAs were predicted to have 81 gene targets, including five genes encoding for transcription factors (CRV2T00000423_1, CRV2T00004800_1, CRV2T00015277_1, CRV2T00000889_1, CRV2T00017200_1), three effectors (CRV2T00014512_1, CRV2T00019066_1, CRV2T00019646_1), four MFS transporters (CRV2T00008216_1, CRV2T00013299_1, CRV2T00013418_1, CRV2T00016330_1), and one protein (CRV2T00007349_1) involved in histone acetylation (Additional file 1: Table S9).

### Gene expression validation using quantitative PCR

RT-qPCR was used to validate RNA-seq data of seven and six transcripts in *C. rosea* and wheat, respectively, during interactions. These transcripts were selected based on their differential expression pattern during Cr-Wr, Δ*dcl1*-Wr, and Δ*dcl2*-Wr compared to respective *C. rosea* and wheat control. The selected *C. rosea* transcripts included CRV2T00011242_1 and CRV2T00009699_1, identified as putative endogenous milRNA gene targets, and CRV2T00016916_1, identified as a cross-species gene target (Table 4). The expression patterns observed by RT-qPCR were in agreement with those obtained by RNA-seq, corroborating the RNAs-seq result (Additional file 2: Fig. S6).

### Trafficking of exogenously applied wheat miRNAs mimics from wheat roots to *C. rosea*

**Fig. 9:**
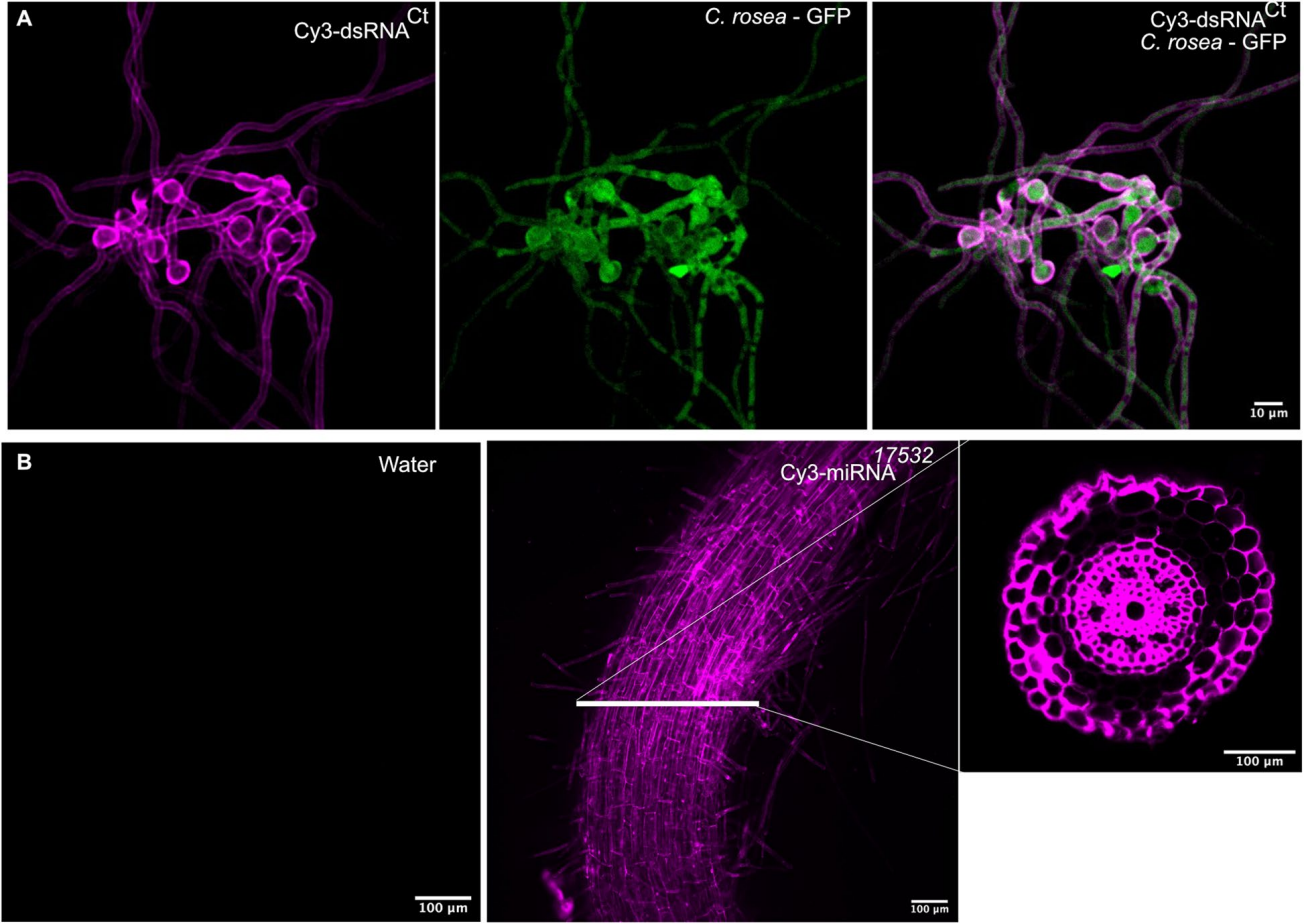
Uptake of dsRNA by *C. rosea* and miRNA mimics by wheat roots. **A** GFP-tagged *C. rosea* conidia (*C. rosea*-GFP) was incubated with Cyanine 3-UTP labeled dsRNA (Cy3-dsRNACt) and examined under a confocal microscope at 24 h post incubation (hpi). Representative confocal microscopy images showing uptake of Cy3-dsRNA by *C. rosea* hyphae and conidia (left panel, magenta), *C. rosea*-GFP control (middle panel), and colocalization of GFP (Green) with Cy3 (Right panel) in Cy3-dsRNACt-treated *C. rosea-*GFP (right panel). **B** Representative confocal microscopy images show the uptake of Cy3-labeled miRNA mimics by wheat roots. The left panel shows a control treatment with no Cy3 fluorescence signal (Magenta). The right panel shows Cy3 miRNA 12061 mimic (Magenta) internalization into wheat roots (confocal microscopy images of root cross sections). For the experiment, artificially synthesized mir_17532_x1 miRNA mimic miR17532 was applied on wheat roots, and a Confocal microscopic analysis of the root surface and horizontal cross-section was performed 24 hpi

To investigate the cross-kingdom trafficking of wheat miRNAs into *C. rosea*, first we determined the ability of *C. rosea* conidia and hyphae to uptake externally applied dsRNA molecules under in vitro conditions. For this, we incubated GFP-tagged *C. rosea* conidia (*C. rosea*-GFP) with Cyanine 3-UTP labeled dsRNA (Cy3-dsRNACt) and examined under a confocal microscope at 24 h post incubation (hpi). The *C. rosea* conidia and hyphae exhibited Cy3 fluorescence (magenta signal) at 561-nm wavelengths, indicating the uptake of dsRNA by *C*. *rosea*. Furthermore, the co-localization of the GFP and Cy3 signals confirmed that *C. rosea* can successfully uptake exogenous dsRNA (Fig. 9A).

We used an in vitro synthesized and Cy3-labeled *Phytophthora infestans* miR8788 mimic (chemically synthesized miRNA molecules used to imitate endogenous miRNAs) to investigate the uptake ability of externally applied miRNA by wheat roots. The miR8788 mimic was spray-inoculated on wheat roots and its uptake was determined 24 (hpi). Water-inoculated roots were used as control treatment. Confocal microscopic analysis of the root surface and horizontal cross-section showed a strong fluorescence signal (magenta), while no signal was observed from the water-treated control, indicating the uptake of miRNA mimics by wheat roots (Fig. 9B).

**Fig. 10:**
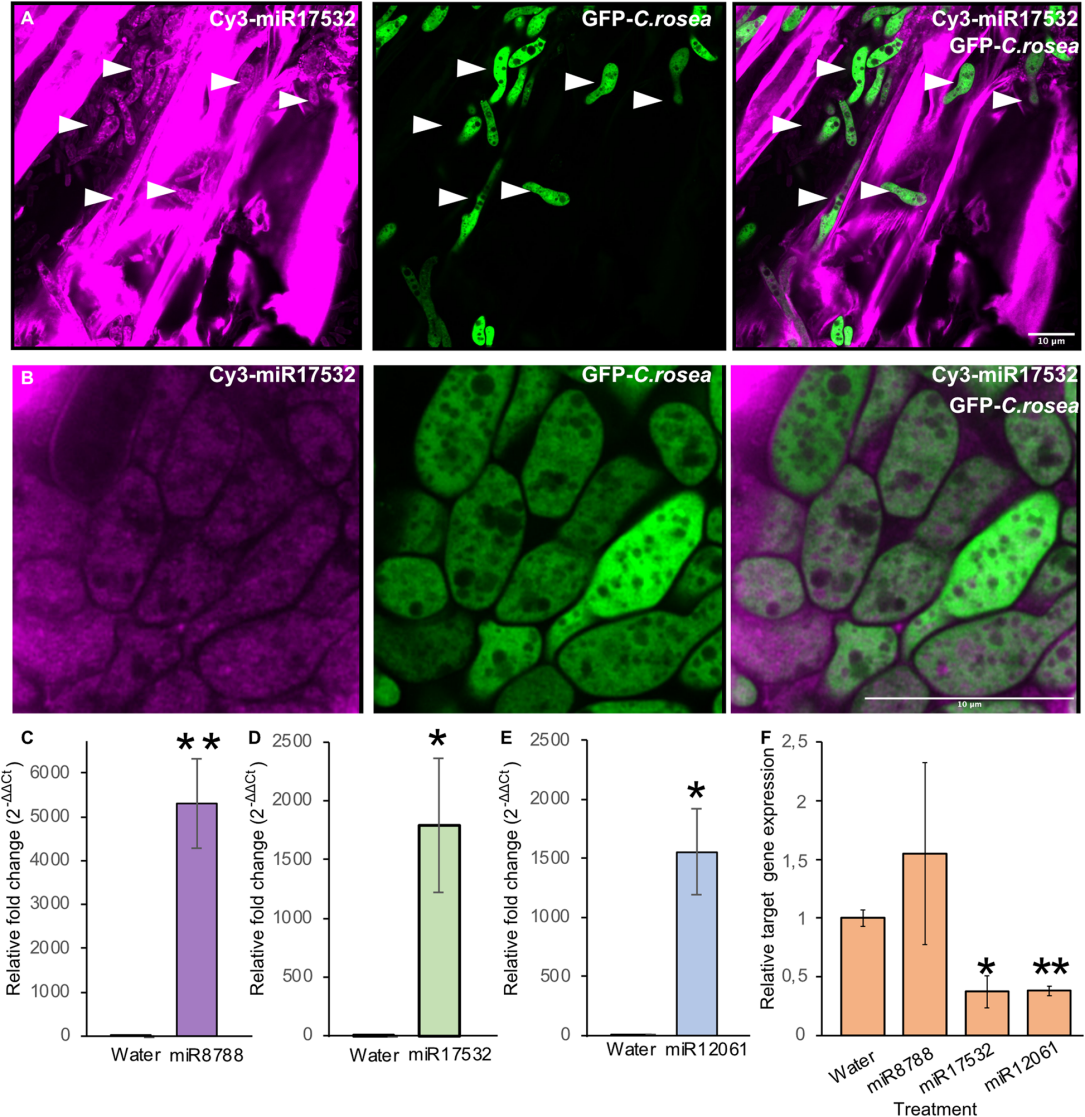
Trafficking of Cy3 labeled wheat mir_17532_x1 mimics (miR17532) from wheat roots to *C. rosea-*GFP conidia and hyphae. **A** Representative confocal images showing the co-localization of Cy3-miR17532 (Magenta), *C*. *rosea-*GFP (Green), and merge (right panel). White arrows indicate germinated *C. rosea* conidia. **B** A representative confocal image (enlarged) shows the internalization of Cy3-miR17532 mimic (Magenta) by *C. rosea* conidia (Green) and submerge (right panel). After 24 h of incubation with miR17532, wheat roots were washed with 0.1M KCl and 0.01 M Triton X100 to remove surface-bound miRNA oligos. Conidia from *C. rosea*-GFP were applied to the roots, and Cy3 fluorescence was determined t72 hpi. **C**–**E** Detection and quantification of wheat miRNAs mimic inside wheat roots using stem-loop-RT-qPCR (**C***P. infestans* miR8788 mimic miR8788; **D** wheat mir_17532_x1 mimic miR17532; **E** wheat mir_12061_x13 mimic miR12061). **F** RT-qPCR showing the expression of the *pks29* gene in *C. rosea* cells after import of miR8788, miR1532, and miR1203 during interaction with wheat roots treated with these miRNA mimics

After determining the internalization of externally applied dsRNAs and miRNAs into *C. rosea* and wheat roots, we investigated the trafficking of sRNAs from wheat roots to *C. rosea*. For this, wheat mir_17532_x1 (miR17532) and mir_12061_x13 (miR12061) mimics were applied to wheat roots. Seedlings inoculated with water or *Phytophthora infestans* miR8788 with no gene targets in wheat roots were used as control treatment. These wheat miRNAs were selected based on their potential cross-kingdom gene target *pks29* in *C. rosea* (Table 4). After confirming the internalization of wheat miRNA mimics into wheat roots by confocal microscopy, we washed the wheat roots with 0.1M KCl and 0.01 M Triton X100 to remove surface-bound miRNA oligos. Subsequently, after applying *C. rosea* spores to the wheat roots, the Cy3 fluorescence was observed in the conidia and hyphae at 72 hpi with no signal in control wheat roots/*C. rosea* hyphae, confirming the transport of the exogenously applied miRNAs from wheat roots to *C. rosea* (Fig. 10A, B, Additional file 2: Fig. S7). To corroborate the internalization of miRNA mimics into wheat roots, we extracted total RNAs from the washed wheat roots and performed stem-loop RT-qPCR using mir_17532_x1, mir_12061_x13 and miR8788 specific primers to quantify miRNA mimics into the wheat roots. The result clearly showed a higher amount of miRNA mimics inside the wheat roots compared to control treatments (Fig. 10C–E).

Since total RNA extracted from wheat roots contained sequences from *C. rosea* genes expressed during interactions, we used these RNAs to validate potential cross-kingdom RNA silencing of *pks29* (CRV2T00016916_1) by wheat miRNAs mir_17532_x1 and mir_12061_x13*.* RT-qPCR analysis revealed a significant reduction in *pks29* expression in *C. rosea* inoculated on wheat roots treated with mir_17532_x1 or mir_12061_x13 mimics compared to control treatments (water and miR8788) (Fig. 10F). These results demonstrate a silencing of the gene in *C. rosea* cells by the wheat miRNAs, providing solid evidence of miRNA trafficking from wheat roots to *C*. *rosea* and suggesting the existence of cross-kingdom RNA silencing between the two organisms.

## Discussion

### Interaction with *C. rosea* induces the upregulation of stress response genes in wheat

Plant-beneficial fungi, for example those belonging to the genus *Trichoderma*, are shown to trigger plant defense response during interaction with the plant host [82, 83]. However, the interplay between the biocontrol fungus *C. rosea* and its host roots remains elusive. In this study, we investigated the interactions between the fungal BCA *C. rosea* and wheat roots, focusing on the transcriptional changes and their regulation that occur during these interactions. We also explored the role of DCLs in regulating gene expression in *C. rosea* during these interactions and its cross-kingdom effect. Previous gene expression studies using qPCR indicated the ability of *C. rosea* to induce the defense response of plant hosts [28, 31, 84, 85]. In the present work, we showed that wheat reacted to the interaction with the fungus by upregulating many stress-resistance genes and downregulating genes involved in cell wall expansion and biosynthesis. We hypothesize that the transcriptional response of wheat is at least partly caused by a *C. rosea* plant cell wall degrading activity. This is supported by the gene enrichment analysis showing *C. rosea* upregulated genes during Cr-Wr were enriched in terms related to polysaccharide catabolism and cell wall modification. As a reaction to *C. rosea-*mediated degradation, wheat reprogramed its genetic machinery for the increased defense-response while, at the same time, downregulating genes associated with development. This growth-defense trade-off is a well-known phenomenon for resource allocation in plants to optimize fitness during host-microbe interactions and stress [86]. This result supports the well-established theory that, at the early stage of interactions, plant defense response transiently decreases, which probably allows colonization of the root to take place. At a later stage, these fungi are perceived as hostile by pattern-recognition receptors. This recognition triggers the plant’s defense response and restricts the fungus to the root’s outermost cell layers [87, 88]. Additionally, at an early stage of interactions, these fungi escape plant defense response by several mechanisms [87, 88].

Many *Trichoderma* spp. share a similar ecological niche to *C. rosea* and are similarly studied for their biocontrol capabilities. Despite this, we observed that the effect of *C. rosea* on wheat roots is quite different to what was observed with *Trichoderma harzianum* by Rubio et al. (2019). The ethylene pathway was induced by both biocontrol agents, with the upregulation of ethylene-responsive transcription factor RAP2-3 like TraesCS1A01G231200.1, and chymotrypsin inhibitors also upregulated during the interaction with both organisms [89]. *T. harzianum* also induced the expression of ethylene-related genes on tomato roots at 72 h post-inoculation, suggesting that the ethylene response is activated in response to different BCAs in multiple plant species [83]. Wound-responsive gene CS2B01G561300.1 was also upregulated in both our work and the one of Rubio et al. (2019), but *T. harzianum* was also able to activate genes linked to the abscisic acid response, while *C. rosea* induced the upregulation of genes encoding for LEA proteins, dehydrins, vicilin-like storage proteins and lectins, and several known disease-resistance proteins. Another difference was that *T. harzianum* induced the downregulation of very few genes (25% compared to the upregulated ones), while in the current study, the number of wheat genes upregulated and downregulated in Cr-Wr was similar. A gene encoding for an expansin (TraesCS4A01G034300.1) was downregulated in both experiments [89], but nine other proteins of the same class were also affected during *C. rosea* interaction with wheat.

Although no studies on the *Trichoderma* transcriptomic response to wheat roots are available, Morán-Diez et al. (2015) studied the reaction of *Trichoderma virens* to maize roots, observing how the fungus upregulated many transporter genes as well as GHs [90]. After prediction with dbCAN2 [91], however, only one of the *T. virens* genes upregulated during the response to maize roots (gene 11696) belonged to the AA9 CAZyme class, which was the one with the most upregulated members in *C. rosea*. *Trichoderma atroviride* also showed differences with *C. rosea* in its CAZyme gene expression during interaction with the roots of plant hosts. In particular, *T. atroviride* was observed to downregulate plant cell wall degrading enzymes before contact with *A. thaliana* roots [92]. On the contrary, in this study we detected similar enzymes being upregulated in *C. rosea* during contact with wheat. This is plausibly related to the interaction stage, as in the current study, root samples were harvested at seven dpi [93]. Additionally, *Clonostachys* spp. were observed to have a higher number of plant cell wall degrading enzymes, like AA9 CAZymes, in their secretome [53], suggesting an essential role of this class of enzymes during the interaction with their plant hosts.

In summary, the results highlighted that the interaction mechanisms between beneficial fungi and host plants depend on the interacting organisms and the experimental conditions. While the activation of the ethylene pathway and of wound-responsive genes were found to be common in response to both *T. harzianum* and *C. rosea*, other phenomena like the expression of genes part of the abscisic acid or the production of LEA proteins are induced by only one of them. The response of the fungus is similarly different, with *C. rosea* upregulating a higher number of CAZymes of AA9 and enzymes involved in plant cell wall degradation.

### Dicer deletion reduces the capability of *C. rosea* to induce defense reactions in wheat

The response of wheat and *C. rosea* to each other appears to be partly sRNA-dependent. Six wheat miRNAs and 15 *C. rosea* milRNAs were downregulated during plant-fungus interaction compared to the control conditions. In comparison, only three plant miRNAs and one fungal milRNAs were upregulated when the two organisms were interacting. This suggests that many milRNAs necessary to regulate the interaction are constitutively expressed in both organisms but downregulated during the interaction, enabling the expression of transcripts that they normally negatively regulate. Most differentially expressed *C. rosea* milRNAs were strongly downregulated in the Δ*dcl2* mutant. Among those, the expression of five was reduced to an undetectable level, while seven milRNAs were unaffected. This suggests either the existence of DCL-independent milRNA synthesis, as is the case for milR-2 in *Neurospora crassa* [94], or a limited complementation activity of DCL1 in the Δ*dcl2* mutant. In any case, DCL-independent milRNA production has previously been reported in *C. rosea* during the interaction with its fungal hosts [21].

The fungal milRNAs downregulated during the interactions were predicted to target 12 transcription factors with opposite expression to the milRNAs, and two of these genes were targeted by DCL2-dependent milRNAs and also showed Spearman anticorrelation between transcript and targeting milRNA significantly higher than the average anticorrelation with other transcripts (Table 4). One of them (CRV2T00000889_1) is a homolog of CreD, reported to be involved in glucose-induced endocytosis and carbon catabolite derepression in *Aspergillus oryzae* [95]. The presence of transcription factors among the milRNA targets suggests that RNA silencing could affect many genes through indirect cascade effects, a hypothesis already advanced in other works [21, 39].

Such indirect effects could explain why, during the interaction between wheat and *C. rosea*, gene expression is so heavily affected by the deletion of the fungal *dcl* genes. We identified 65 wheat stress-responsive genes upregulated in Cr-Wr but not in either Δ*dcl1*-Wr or Δ*dcl2*-Wr, suggesting an essential role of DCL-dependent gene expression regulation to induce plant defense genes (Fig. 6). This result correlates well with the findings of our previous paper, in which *C. rosea* Δ*dcl2* was observed to have a reduced capacity to control *Fusarium* foot rot in wheat plants [21]. Such defense reactions could be caused by *C. rosea* plant cell wall degrading enzymes, many of which are upregulated in Cr-Wr but not in Δ*dcl1*-Wr or Δ*dcl2*-Wr, releasing fragments acting as damage-associated molecular patterns. This aligns with the other results from the transcriptome analysis, which point to *C. rosea* WT inducing defense reactions in wheat through limited plant cell wall degradation. The lack of upregulation of plant defense genes in wheat root was coupled with the lack of downregulation of genes associated with cell wall loosening, expansion and permeabilization, which were downregulated in Cr-Wr but not in either Δ*dcl1*-Wr or Δ*dcl2*-Wr. This includes genes coding for expansin-like proteins and a peroxidase-57, whose overexpression increases cuticle permeability in *A. thaliana* [75]. We speculate that the downregulation of genes associated with plant cell expansion, and upregulation of genes involved in defense responses, both happening in Cr-Wr but not in the mutants, resulted in increased root colonization by Δ*dcl2* mutant compared to *C. rosea* WT. However, other possible mechanisms of increased root colonization by Δ*dcl2* strains cannot be ruled out.

Investigating instead putative direct RNA silencing, among the targets predicted for DCL2-dependent milRNAs and supported by target prediction, opposite expression, and Spearman anticorrelation significantly higher than the one with the average transcript, we found one 3.A.1.208 ABC transporter (CRV2T00011673_1), a class responsible for multidrug resistance in cancer cells [96]. It has been observed in previous studies that the upregulation of transporters is an integral part of *C. rosea*’s response to plant pathogens [36], probably as a protection mechanism against mycotoxins and other harmful compounds [26, 97]. Another target is cytochrome P450 monooxygenase CRV2T00011242_1, also belonging to a class of proteins with a role in xenobiotic detoxification [98, 99]. Other two multidrug transporters, belonging to MFS classes 2.A.1.2 (CRV2T00004939_1) and 2.A.1.3 (CRV2T00009699_1), were targeted by milRNAs cro-mir-30 and cro-mir-76, whose expression was not DCL2-dependent, and one of them (CRV2T00004939_1) was observed to be involved in *C. rosea* response to *B. cinerea* and *F. graminearum* [36]*.* While there is no information available on the role of these transporters and cytochrome P450 monooxygenase in *C. rosea*-wheat interactions, upregulation of these genes is reported in *T. asperellum* and *T. asperelloides* during the interactions with plant hosts [88, 100]. The upregulation of these genes indicates their potential roles in tolerating xenobiotic compounds that may come from the plant hosts during interactions. Highly supported targets of non-DCL2 dependent milRNAs also included two acid phosphatases (CRV2T00005739_1 and CRV2T00021953_1), proteins known to induce salt resistance in *A. thaliana* during interaction with *T. atroviride* [101] (Table 4)*.* This suggests that Dicer-dependent and independent RNA silencing mechanisms regulate gene expression during *C. rosea* interaction with wheat and that the genes directly targeted by milRNAs affect regulation, *C. rosea* biocontrol activity, and wheat stress resistance.

### Evidence of wheat miRNAs plausibly affect the expression of *C. rosea* genes through cross-kingdom RNA silencing

Genes of interest were also targeted by wheat miRNAs upregulated during the plant-fungus interaction. In particular, wheat mir_15432_x2 was predicted to target a mixed-linked glucan synthase eight involved in cellulose synthesis (TraesCS1D01G107100.1), suggesting wheat uses RNA silencing to reduce cellulose production during the interaction with *C. rosea*. This reduction could facilitate *C. rosea* colonization of wheat roots. A similar mechanism has been observed in *A. thaliana,* with mutants with inactive RNA silencing showing less root colonization by biocontrol agent *T. atroviride* [24]. Moreover, the same miRNA (mir_15432_x2) was also identified as upregulated in the wheat cultivar Zhengyin 1 after dehydration stress, with the ID “wheat-miR-683” [102]. On the fungal side, however, only the milRNA cro-mir-73 was more expressed in the plant-fungal interaction than in the control. It had only three putative targets, none showing significant anti-correlation with the targeting milRNA.

The silencing of *dcl* genes in *C. rosea* altered the expression pattern of wheat genes, and the plant reacted to the mutants by producing different miRNAs compared to how it responded to the WT. Wheat miRNA mir_15848_x2, for example, was upregulated in response to the Δ*dcl1* mutant, and it was predicted to target the ABC transporter TraesCS3D01G258300.1. Three wheat miRNAs (mir_17532_x1, mir_16010_x2 and mir_12061_x13) are also predicted to be involved in cross-kingdom RNA silencing. All of them are downregulated during Cr-Wr, and they are predicted to target the *C. rosea* transcript CRV2T00016916_1, significantly upregulated (log2FC = 5.63) during Cr-Wr compared to control *C. rosea*. These three miRNAs were not downregulated during the Δ*dcl1*-Wr interaction, and the upregulation of CRV2T00016916_1 is significantly lower in that condition (log2FC = 4). This transcript encodes the polyketide synthase PKS29, producing an unknown compound required for antagonism against *B. cinerea* and for biocontrol of fusarium foot rot on barley [35]. Unfortunately, the effect of this unknown compound in *C. rosea*-wheat interactions is currently unknown. However, we can speculate that these wheat miRNAs were downregulated during Cr-Wr to induce the production of antimicrobial compounds by *C. rosea*, benefitting wheat in the fight against plant pathogens. The fluorescence experiment using externally applied miRNAs mimics miR17532 and miR12061 demonstrated that these wheat mimics can indeed enter *C. rosea* cells during interactions and silence the expression of *pks29*. While certain proof of direct targeting of the *pks29* transcript by these miRNAs is still missing, as the downregulation of the gene could be the result of other effects on gene regulation caused by the same miRNAs, our study provides preliminary solid evidence towards the possibility of cross-kingdom RNA silencing activity between wheat and *C. rosea*. More experimental evidence is needed to corroborate miRNAs trafficking from wheat roots to *C. rosea* and cross-kingdom RNA silencing.

## Conclusions

**Fig. 11:**
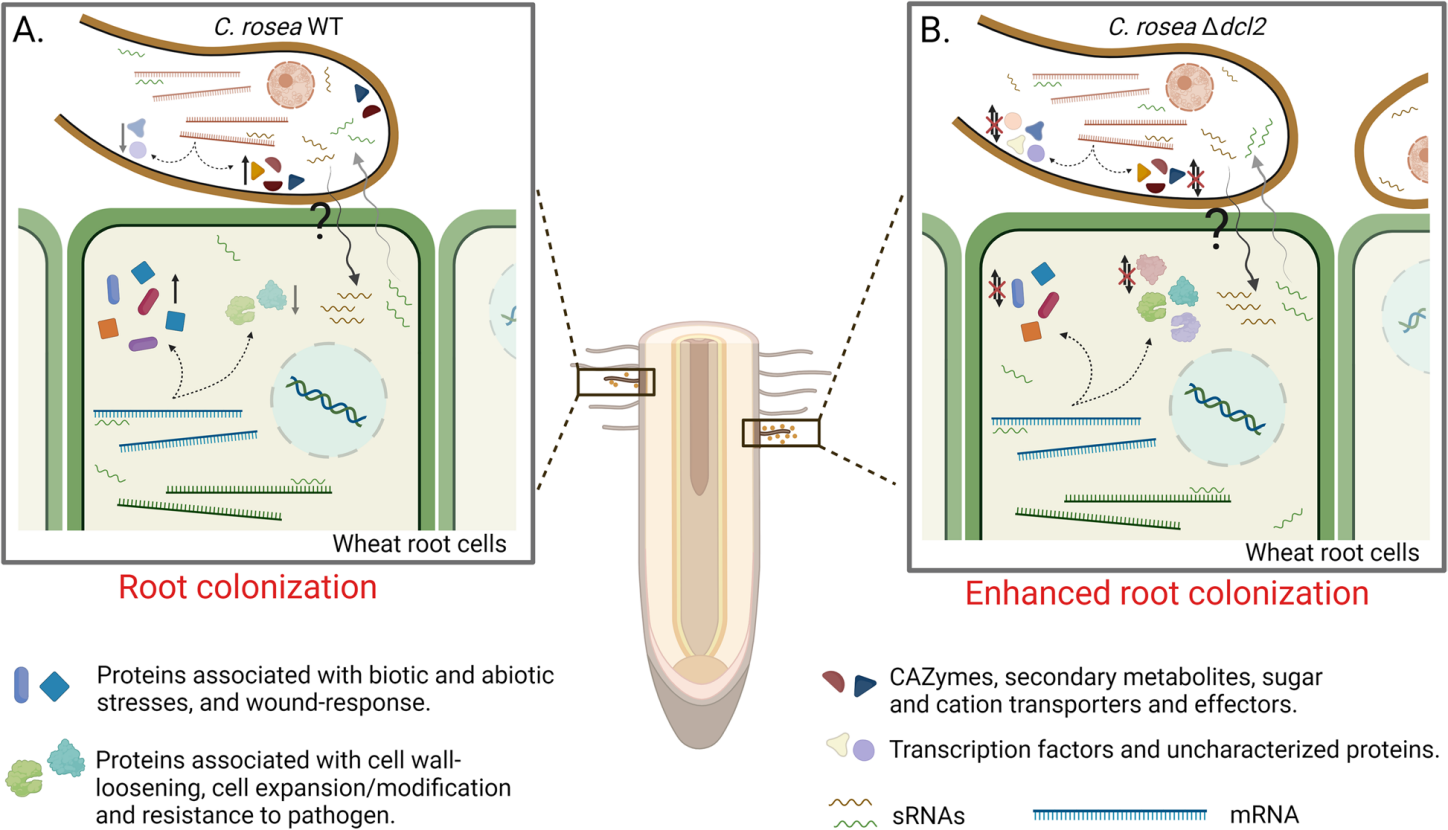
Illustration of molecular dialogue and cross-kingdom RNA silencing between *C. rosea* and wheat. **A** The *Clonostachys rosea*-wheat interaction triggers the upregulation of wheat genes associated with biotic and abiotic stress tolerance and the downregulation of genes involved in cell wall loosening and expansion, leading to controlled colonization of wheat roots by *C. rosea*. Concurrently, genes related to carbohydrate catabolism, transport, and effector production are upregulated in *C. rosea*. **B** During the interaction between *Δdcl2* and wheat roots, the expression patterns of many of these genes in wheat and *C. rosea* are altered, resulting in enhanced root colonization by *C. rosea*. The regulation of these gene expression patterns in wheat and *C. rosea* is potentially mediated by small RNAs at both endogenous and cross-kingdom levels. ↑ Indicates gene upregulation, ↓ indicates gene downregulation, ? Indicate lack of experimental validation for sRNA movement from *C*. *rosea* to wheat roots

In conclusion, the interaction between wheat and the biocontrol fungus *C. rosea* is a complex and dynamic process that involves the expression of numerous genes and the regulation of miRNAs in both the plant and the fungus. This interaction triggers a cascade of molecular events in wheat, leading to the activation of stress resistance genes and the modulation of cell wall-related processes, suggesting a trade-off between defense and growth. *Clonostachys rosea*, in turn, showed altered expression of genes involved in carbohydrate catabolism, membrane transport, and the production of effector molecules (Fig. 11). In addition, the study explores the effects of DCL (Dicer-like) gene deletions in *C. rosea* and their impact on root colonization as well as the transcriptomic responses of both *C. rosea* and wheat during their interactions. Notably, deleting DCL genes in *C. rosea* alters the gene expression pattern in wheat. This results in a lack of upregulation of genes associated with stress resistance, including LEA proteins, dehydrins, wound-response proteins, and LRR receptors (Fig. 11). Deletion of *dcl2* had a more pronounced effect on wheat gene expression than *dcl1* deletion, which aligns with the increased root colonization ability of DCL2 deletion strains. The study identified candidate miRNAs in wheat and milRNAs in *C. rosea* and their potential endogenous and cross-kingdom gene targets. These were differentially expressed during their interactions, suggesting a complex network of sRNA-mediated gene regulation, and they include transcription factors like a CreD homolog as well as MFS and ABC transporters. Interestingly, wheat miRNAs were also predicted to be able to affect the expression of *C. rosea pks29*, which is involved in the production of a compound with antifungal activity. Moreover, the externally applied mimics of these wheat miRNAs were shown to be transported to *C. rosea* from wheat roots and can silence the expression of *pks29* (Fig. 11). This suggests the plant could control the *C. rosea* production of antimicrobial compounds through cross-species gene silencing. Furthermore, this study underscores the importance of sRNAs as crucial players in the intricate molecular interplay between plant and fungal species used for biocontrol, potentially regulating gene expression at both endogenous and cross-kingdom levels. Further exploration of these sRNA-mediated mechanisms and functional characterization of candidate fungal miRNAs and plant miRNAs and their potential gene targets can provide valuable insights into the beneficial fungus-plant interactions relevant to plant health promotion and biocontrol. It may help develop novel strategies for enhancing plant resistance to biotic and abiotic stressors in agriculture, improving plant growth and optimizing plant health.

## Methods

### Root colonization assay

An experimental setup was done as described previously [28] to quantify the root colonization. Root colonization was determined five days post inoculation (dpi) by quantifying the DNA level of *C*. *rosea* strains in wheat roots using qPCR [103]. The actin gene *act* was used as the target gene for *C*. *rosea*, and *Hor1* [28] was used as the target gene for wheat [104]. Root colonization was expressed as the ratio between act and Hor1. The experiment was performed in five biological replicates, each consisting of five seedlings. Analysis of variance (ANOVA) was performed using a general linear model approach implemented in Statistica version 14 (TIBCO Software Inc., Palo Alto, CA, United States). Pairwise comparisons were made using Fisher’s method at the 95% significance level.

### Sample preparation for RNA sequencing

Surface sterilized wheat seeds of the cultivar “Stava” were germinated on sterilized moist filter paper placed in 9-cm-diameter petri plates (five seeds per plate) following the procedures described before [28]. Three-day-old wheat seedlings were inoculated by dipping the roots for 3 min in *C. rosea* IK726 spore suspensions (1 × 10^7^ spore/ml) in sterile conditions, transferred back to the filter paper in Petri plates, and incubated at 20 °C as described before [28]. Roots were harvested at seven dpi and snap-frozen in liquid nitrogen. Water-inoculated wheat roots were used as a control for the wheat transcriptome, while *C. rosea* grown on moist filter paper was used as a control for the *C. rosea* transcriptome. The experiment was performed in three biological replicates, with five seedlings per replicate for each treatment.

### RNA extraction library preparation and sequencing

Total RNA was extracted using the mirVana miRNA isolation kit following the manufacturer’s protocol (Invitrogen, Waltham, MA). The RNA quality was analyzed using a 2100 Bioanalyzer Instrument (Agilent Technologies, Santa Clara, CA), and concentration was measured using a Qubit fluorometer (Life Technologies, Carlsbad, CA). The library was prepared for sRNA and mRNA sequencing, and paired-end sequencing was conducted at the National Genomics Infrastructure (NGI) in Stockholm, Sweden. The sRNA library was generated using the TruSeq small RNA kit (Illumina, San Diego, CA), while the mRNA library was generated using the TruSeq Stranded mRNA poly-A selection kit (Illumina, San Diego, CA). The sRNA and mRNA libraries were sequenced on one NovaSeq SP flowcell with a 2 × 50 bp reads and NovaSeqXp workflow in S4 mode flow cell with 2 × 151 setup, respectively, using Illumina NovaSeq6000 equipment at NGI Stockholm. The Bcl to FastQ conversion was performed using bcl2fastq_v2.19.1.403 from the CASAVA software suite [105]. The quality scale used was Sanger/phred33/Illumina 1.8 + . Three biological replicates were sequenced for both transcriptome and sRNAs for each of the analyzed conditions: wheat roots, *C. rosea* WT growing in PDB media, *C. rosea* WT interacting with wheat roots (Cr-Wr), *C. rosea* Δ*dcl1* interacting with wheat roots (Δ*dcl1*-Wr), *C. rosea* Δ*dcl2* interacting with wheat roots (Δ*dcl2*-Wr).

### Mapping and differential expression analyses

Adapter and quality trimming were conducted for sRNA and mRNA reads using bbduk v. 38.9 [106], and quality was then checked using fastqc [107]. The options used for bbduk were as follows: ktrim = r k = 23 mink = 11 hdist = 1 tpe tbo qtrim = r trimq = 10.

For mRNAs, reads were mapped to the *C. rosea* IK726 genome (GCA_902827195) and the IWGSC “Chinese Spring” genome assembly [108] using the splice-aware aligner STAR [109] with default parameters and the option “–outFilterMultimapNmax 40”. Reads mapping to both genomes were excluded with an ad hoc pipeline using samtools v. 1.9 [110] and Picard tools v. 2.18.29 (http://broadinstitute.github.io/picard/). The number of reads mapping to each gene was then evaluated using featureCounts v. 2.0.1 [111], and differential expression was determined with the DESeq2 R package v. 1.28.1 [112] using a minimal threshold of 1.5 for log2(FC) and 0.05 for FDR adjusted *p*-value.

Normalized expression values were obtained from DESeq2 and used to perform a co-expression analysis with WCGNA [113] using only differentially expressed genes. The soft-thresholding power was 6 for *C. rosea* genes and 16 for wheat genes, and the function “binarizeCategoricalVariable” was used to convert the categorical variables into numerical ones.

BLAST2GO [114] was used to determine enriched GO terms in the differentially expressed genes, using a Fisher test corrected with the FDR method. The adjusted *p* value threshold was set at 0.05, and enriched biological processes were visualized using Python seaborn v. 0.12.2 [115] and Scientific Inkscape (https://github.com/burghoff/Scientific-Inkscape).

Reformat.sh v. 38.9 [106] was used only to retain sRNA reads between 18 and 32 bp in length, and rRNAs, tRNAs, snRNAs, and snoRNAs were removed from the dataset using SortMeRNA v. 4.2.0 [116] using as references RNA sequences downloaded from SILVA and the NRDR database [117, 118]. The filtered sRNA reads were then mapped to the *C. rosea* and wheat transcriptomes using bowtie v. 0.12.9 [119] using the following options:

-S -k 101 -n 2 -l 18 -m 200 –best –strata

In the case of wheat, only High Confidence transcripts (https://urgi.versailles.inra.fr/download/iwgsc/IWGSC_RefSeq_Annotations/v1.0/) were used, and the options “-n 3” were added to compensate for the fact that the sequenced genome was of a different cultivar from the one used for the experiment.

FeatureCounts v. 2.0.1 was used to quantify the reads mapping to each transcript, only counting antisense mappings, and normalized sRNA-mapping values were obtained for each gene using DESeq2 [112].

Prediction of co-expression modules was carried out using the R package WGCNA [49]. The analysis was performed twice: once using the normalized expression values of differentially expressed wheat genes and the other using the same values for *C. rosea* genes. In this way, we obtained separate modules for wheat and *C. rosea* genes.

### Functional annotation

The function of *C. rosea* genes was determined according to functional annotation performed in previous publications [21, 53], while differentially expressed wheat genes had their domains predicted with InterProScan [120], and they were compared with the NCBI database through BLAST [121]. Additionally, effectorP was used to predict *C. rosea* effector-like proteins [122].

### milRNA prediction, differential expression, and target prediction

Known milRNA sequences of *C. rosea* were retrieved from previous publications [21], while known wheat miRNAs were retrieved from the Wheat miRNA web portal and MiRbase [123, 124]. Novel milRNAs of *C. rosea* were predicted with MiRDeep2 v. 2.0.1.3 using default parameters [125], while novel wheat miRNAs were predicted using MiRDeep-P2 v. 1.1.4 [126].

The presence of each milRNA/miRNAs was quantified in each sample by counting their occurrence in the clean reads file, allowing for one mismatch using agrep [127].

Target prediction was made using the plant-based tools psRNATarget, Targetfinder, psROBOT, and TAPIR, using the latter two through the sRNA toolbox [59–63], and milRNA-target couples predicted by at least two tools were retained. Self-targets of *C. rosea* milRNAs were also predicted with the animal-based tools PITA, Miranda, TargetSpy, and simple seed analysis, all used through the sRNA toolbox, and milRNA-target couples predicted by at least three tools were retained [128–131]. Afterwards, we only retained milRNA-target couples showing opposite expression between the milRNA and the putative target (if one was upregulated in a specific condition, then the other needed to be downregulated). For this filtering step, we used DESeq2 v. 1.28.1 [112] with a minimal threshold of 1.5 for log2(FC) and 0.05 for FDR-adjusted *p*-value for milRNAs, while for putative targets, we set a threshold of 0.05 for FDR adjusted *p*-value but no threshold for log2(FC). Additionally, for each target, we calculated the Spearman and Pearson correlation between the miRNA counts and the target mRNA counts, and we compared it to the average correlation of the miRNA with any other transcript of the same organism. As done in a different study [132], we used the Wilcoxon rank sum test with a *p*-value threshold of 0.1 to determine if the anti-correlation between milRNA and target was significantly higher than the one between the milRNA and the average transcript.

### In vitro assay to visualize uptake of dsRNA by *C. rosea*

To determine the ability of *C. rosea* conidia and hyphae to uptake dsRNA molecules, Cyanine 3-UTP (Enzo Life Sciences, East Farmingdale, NY) labeled dsRNA (Cy3-dsRNACt) provided in the MEGAscript RNAi kit was synthesized following the protocol from the manufacturer (Invitrogen, Waltham, MA). Ten micrograms of Cy3-dsRNACt were mixed with conidial suspension (1 × 10^6^) harvested from *C*. *rosea* strain IK726 tagged with GFP (*C*. *rosea* IK726-GFP) in a 1.5-ml microcentrifuge tube. After 24 h of incubation at 19 °C, the uptake of Cy3-dsRNACt into the germinating conidia and hyphae was visualized using an LSM880 confocal microscope (Zeiss Microscopy, Jena, Germany) with lasers at 488 and 561 nm wavelengths exciting GFP and Cy3, respectively. The experiment was performed in three biological replicates.

### Exogenous application of miRNAs mimics to wheat roots

Surface-sterilized wheat seeds were germinated on moist filter paper in sterile Magenta vessels within a Panasonic MLR-352 PE Climate (Plant Growth) Chamber at 25 °C temperature and 85% relative humidity. Three-day-old seedlings were inoculated with 1 μM of fluorescent Cy3-labeled miRNA mimics miR12061 and miR17532. Seedlings inoculated with water or *P. infestans* miR8788 with no gene targets in wheat roots were used as control treatment. These miRNA mimics were custom synthesized using Merck’s custom oligo designing platform (Merck, USA). The sequences were modified to contain Cy3 fluorophore at the 5′ end and 2′-O-methyl modification for the base at the 3′ end (Additional file 1: Table S10). The seedlings were washed with 0.M KCl and 0.01 M Triton X100 post 24 hpi to remove surface-bound miRNAs oligos. They were inoculated with a *C. rosea*-GFP spore suspension (1 × 10^7^ spores/ml) and incubated in sterile 1/2 MS liquid media for 24 h. Roots were harvested 24 hpi and were divided into two halves. One half was prepared for confocal microscopy, while another half was snap-frozen in liquid nitrogen for RNA extraction. Total RNA was extracted using the mirVana miRNA isolation kit following the manufacturer’s protocol (Invitrogen, Waltham, MA). The experiment was performed in three biological replicates, each with five seedlings per replicate.

### RT-qPCR and stem-loop RT-qPCR analysis

After DNaseI (Fermentas, St. Leon-Rot, Germany) treatment, 1 µg of total RNA was reverse transcribed using the iScript cDNA synthesis kit (BioRad, Hercules, CA). Transcript levels were quantified by RT-qPCR using the SYBR Green PCR Master Mix (Fermentas, St. Leon-Rot, Germany) and gene-specific primer pairs presented in Additional file 1: Table S10 in an iQ5 qPCR System (Bio-Rad, Hercules, CA) as described previously. For gene expression analysis in *C*. *rosea*, relative expression levels for the target gene in relation to β-tubulin gene [30] were calculated from threshold cycle (Ct) values using the 2^−ΔΔCt^ method (Livak and Schmittgen, 2001). For gene expression analysis in wheat roots, expression data were normalized to expression of the wheat β-tubulin gene [23]. Gene expression analysis was carried out in three biological replicates, each based on two technical replicates. Quantitative stem-loop qRT-PCR was performed with the CFX96 real-time PCR detection system (Bio-Rad) using the SYBR Green mix (Bio-Rad) following the protocol previously described [21]. For steam-loop RT-qPCR, relative expression levels of miRNA mimics in relation to the GAPDH gene were calculated from threshold cycle (Ct) values using the 2^−ΔΔCt^ method.

## Supplementary Information

Additional file 1: Table S1: Sample-by-sample mRNA and sRNA mapping results on wheat and *C. rosea*. Table S2: Wheat and *C. rosea* transcripts differentially expressed during interactions in this study. Analysis carried out through DESeq2 v. 1.28.1 with default parameters. The adjusted *p*-value threshold was fixed at 0.05, and minimum log2(FC) was set at 1.5. Table S3: Gene ontology terms (GOs) enriched in wheat and *C. rosea* genes upregulated or downregulated during Cr-Wr. The analysis was done with BLAST2GO, using a Fisher test corrected with the FDR method. The adjusted *p*-value threshold was set at 0.05. Table S4: Wheat genes of interest upregulated or downregulated during the interaction with *C. rosea* WT. Table S5: The top 20 highly upregulated or downregulated wheat genes during *C. rosea*-wheat interactions compared to the wheat control. Table S6: Differentially expressed wheat genes with a role in cell wall synthesis or modification, resistance, or induction of defense reactions, as well as *C. rosea* CAZymes or effectors. All the genes were upregulated or downregulated in Cr-Wr but not in Δ*dcl1*-Wr and Δ*dcl2*-Wr. Log2Fc values are in bold when significant (adjusted *p*-value < 0.05). Table S7: The top 20 highly upregulated wheat genes or downregulated *C. rosea* genes during the interactions with wheat roots*.* Table S8: Sequence, length, and expression level of detected expressed milRNAs. Note that in this analysis, the conditions “Δ*dcl1*-Wr” and “Δ*dcl2*-Wr” were compared with “Cr-Wr” and not to the control in the differential expression analysis. This way, the conditions involving mutants (Δ*dcl1*-Wr and Δ*dcl2*-Wr) were compared directly with the same condition with the WT (Cr-Wr) rather than with *C. rosea* in vitro or non-inoculated wheat. Table S9: Transcripts predicted to be targeted by putative milRNAs. At least two target prediction tools have predicted all putative targets, and they show opposite expressions to the targeting milRNAs (a target needs to be upregulated when the targeting milRNA is downregulated). Note that in this analysis, the conditions “Δ*dcl1*-Wr” and “Δ*dcl2*-Wr” were compared with “Cr-Wr” and not to the control in the differential expression analysis. This way, the conditions involving mutants (Δ*dcl1*-Wr and Δ*dcl2*-Wr) were compared directly with the same condition with the WT (Cr-Wr) rather than with *C. rosea* in vitro or non-inoculated wheat. Table S10: List of primers used in this study.

Additional file 2: Fig. S1: Gene ontology terms referring to biological processes enriched in wheat genes or *C. rosea* genes differentially expressed during the interaction between the two organisms. The analysis was done with BLAST2GO, using a Fisher test corrected with the FDR method. The adjusted pvalue threshold was set at 0.05, and enriched biological processes were visualized using Python seaborn v. 0.12.2 and Scientific Inkscape (https://github.com/burghoff/Scientific-Inkscape). The heatmap shows the negative LOG_10_ of the FDR-corrected *p*-value obtained in a Fisher test to calculate gene ontology enrichment. Fig. S2: The heatmap shows the Spearman correlation between the module eigengenes of co-expression modules generated with WGCNA and the conditions examined in this study. Wheat roots (Wheat Control), *C. rosea* WT interacting with wheat roots (Cr-Wr), *C. rosea* Δ*dcl1* interacting with wheat roots (Δ*dcl1*-Wr), *C. rosea* Δ*dcl2* interacting with wheat roots (Δ*dcl2*-Wr). The modules were generated using the normalized expression values of differentially expressed wheat genes. Asterisks indicate significant correlation or anticorrelation. Fig. S3: The heatmap shows the Spearman correlation between the module eigengenes of co-expression modules generated with WGCNA and the conditions examined in this study. *C. rosea* WT growing in PDB media (Cr Control), *C. rosea* WT interacting with wheat roots (Cr-Wr), *C. rosea* Δ*dcl1* interacting with wheat roots (Δ*dcl1*-Wr), *C. rosea* Δ*dcl2* interacting with wheat roots (Δ*dcl2*-Wr). The modules were generated using the normalized expression values of differentially expressed *C. rosea* genes. Asterisks indicate significant correlation or anticorrelation. Fig. S4: The figure contains information regarding the wheat miRNAs detected in this study. A: length distribution. B: differential expression. C: number of putative gene targets showing inverse expression pattern with the miRNAs. Fig. S5: The figure contains information regarding the *C. rosea* milRNAs detected in this study. A: length distribution. B: differential expression. C: number of putative gene targets showing an inverse expression pattern with the milRNAs. Fig. S6: Gene expression validation by RT-qPCR. Expression profiles of selected *C. rosea* (A) and wheat (B) genes were analyzed during interactions. Relative expression levels in *C. rosea* and wheat were normalized by respective *C. rosea* and wheat β-tubulin (TUB) expression and presented in relation to non-interaction control. Error bars represent standard deviation based on three biological replicates. Different letters indicate statistically significant differences (*p* < 0.05) based on Fisher’s exact test. The table highlighted in green indicates gene expression patterns from RNAseq. *Indicates endogenous gene targets (*C. rosea* genes targeted by *C. rosea* milRNAs, see Table 4), # indicates cross-kingdom gene targets (*C. rosea* gene targeted by three wheat miRNAs mir_17532_x1, mir_16010_x2, mir_12061_x13 (see Table 4). Fig. S7: Trafficking of Cy3 labeled wheat mir_12061_x13 mimics (miR17532) (A) and *Phytophthora infestans* miR8788 (B) from wheat roots to *C. rosea-*GFP conidia and hyphae A. Representative confocal images showing the co-localization of Cy3-miR17532 (Magenta), *C*. *rosea-*GFP (Green) and merge (right panel). B. Representative confocal images showing the co-localization of Cy3-miR8788 (Magenta), *C*. *rosea-*GFP (Green) and merge (right panel). Twenty-four hours post incubation (hpi) with the mimics, wheat roots were washed with 0.M KCl and 0.01 M Triton X100 to remove surface-bound miRNA oligos. Conidia from *C. rosea*-GFP were applied to the roots, and Cy3 fluorescence was determined 72 hpi.

## Data Availability

All the sequencing data generated in this study is available at the European Nucleotide Archive under Bioproject PRJEB64581.

## Abbreviations

A: Adenine
AA9: Auxiliary activity family 9
ABA: Abscisic acid
ABC: Adenosine tri phosphate
ADP: Adenosine di phosphate
AGO: Argonaute
BCA: Biocontrol agent
C: Cytosine
CAZymes: Carbohydrate-active enzymes
Cr: *Clonostachys rosea*
Cy3: Cyanine3
DCL: Dicer like
DNA: Deoxyribonucleic acid
G: Guanidine
GFP: Green fluorescent protein
GH: Glucosyl hydrolase
GO: Gene ontology
KCL: Potassium chloride
LEA: Late embryogenesis abundant
MFS: Major facilitator superfamily
milRNA: MicroRNA-like RNA
miRNA: MicroRNA
mRNA: Messenger RNA
NBS-LRR: Nucleotide-binding site–leucine-rich repeat
PCR: Polymerase chain reaction
PKS: Polyketide synthase
PTGS: Post-transcriptional gene silencing
RNA: Ribonucleic acid
ROS: Reactive oxygen species
RT-qPCR: Real-time quantitative polymerase chain reaction
siRNAs: Short interference RNA
sRNA: Small RNA
U: Uridine
UTP: Uridine triphosphate
Wr: Wheat root
WT: Wildtype
