# Supplementary material for: RNA silencing is a key regulatory mechanism in the biocontrol fungus Clonostachys rosea-wheat interactions
Source: BMC Biol. 2024 Sep 30;22:219. doi: 10.1186/s12915-024-02014-9 (PMC11441109; doi:10.1186/s12915-024-02014-9)
Supplement: Supplementary file 2 — Additional file 2: Fig. S1: Gene ontology terms referring to biological processes enriched in wheat genes or C. rosea genes differentially expressed during the interaction between the two organisms. The analysis was done with BLAST2GO, using a Fisher test corrected with the FDR method. The adjusted pvalue threshold was set at 0.05, and enriched biological processes were visualized using Python seaborn v. 0.12.2 and Scientific Inkscape (https://github.com/burghoff/Scientific-Inkscape). The heatmap shows the negative LOG10 of the FDR-corrected p-value obtained in a Fisher test to calculate gene ontology enrichment. Fig. S2: The heatmap shows the Spearman correlation between the module eigengenes of co-expression modules generated with WGCNA and the conditions examined in this study. Wheat roots (Wheat Control), C. rosea WT interacting with wheat roots (Cr-Wr), C. rosea Δdcl1 interacting with wheat roots (Δdcl1-Wr), C. rosea Δdcl2 interacting with wheat roots (Δdcl2-Wr). The modules were generated using the normalized expression values of differentially expressed wheat genes. Asterisks indicate significant correlation or anticorrelation. Fig. S3: The heatmap shows the Spearman correlation between the module eigengenes of co-expression modules generated with WGCNA and the conditions examined in this study. C. rosea WT growing in PDB media (Cr Control), C. rosea WT interacting with wheat roots (Cr-Wr), C. rosea Δdcl1 interacting with wheat roots (Δdcl1-Wr), C. rosea Δdcl2 interacting with wheat roots (Δdcl2-Wr). The modules were generated using the normalized expression values of differentially expressed C. rosea genes. Asterisks indicate significant correlation or anticorrelation. Fig. S4: The figure contains information regarding the wheat miRNAs detected in this study. A: length distribution. B: differential expression. C: number of putative gene targets showing inverse expression pattern with the miRNAs. Fig. S5: The figure contains information regarding the C. rosea [file 12915_2024_2014_MOESM2_ESM.pdf]

Additional File 2: Figure S1

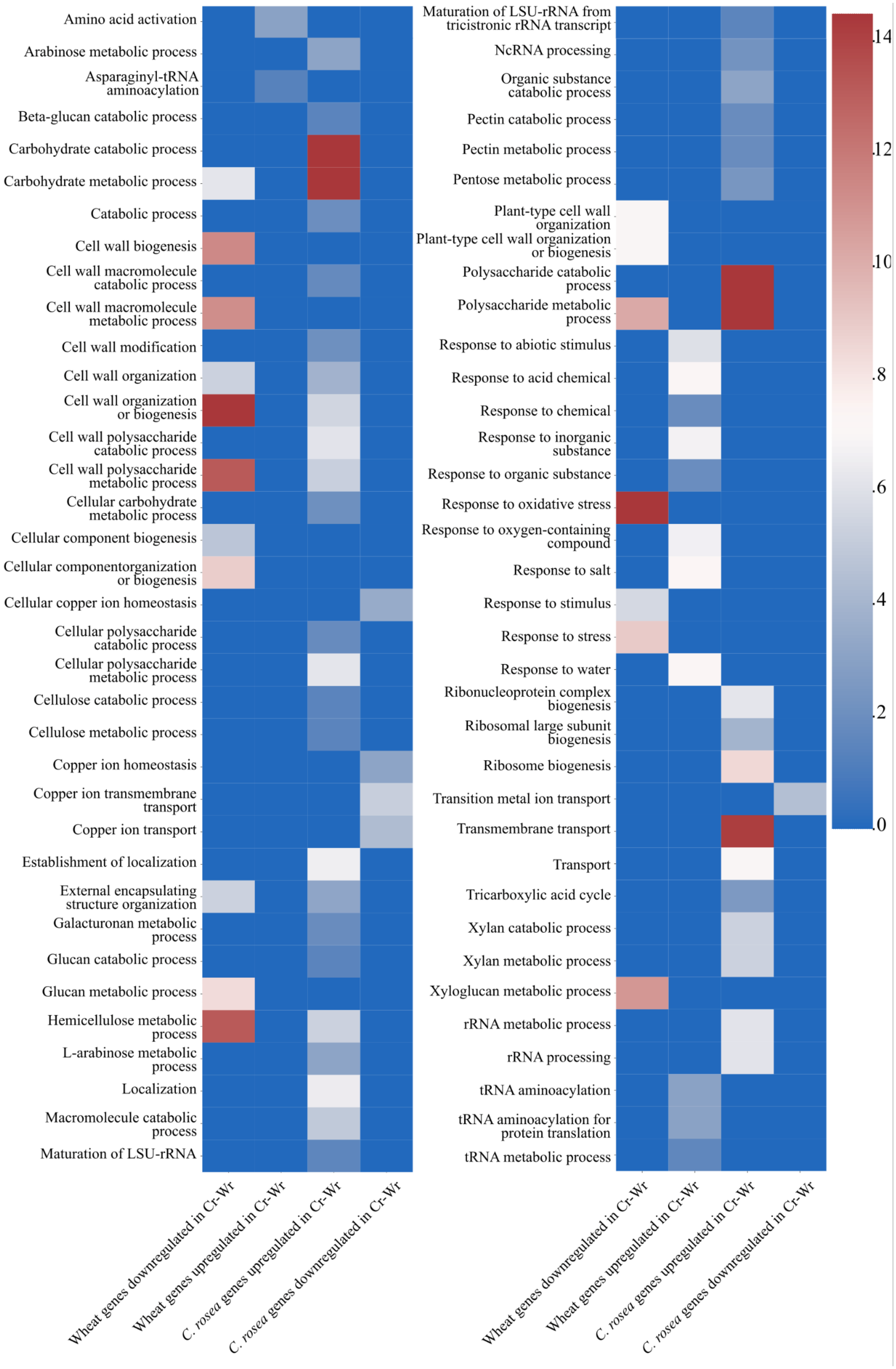

Figure S1: Gene ontology terms referring to biological processes enriched in wheat genes or *C. rosea* genes differentially expressed during the interaction between the two organisms. The analysis was done with BLAST2GO, using a Fisher test corrected with the FDR method. The adjusted pvalue threshold was set at 0.05, and enriched biological processes were visualized using Python seaborn v. 0.12.2 and Scientific Inkscape (https://github.com/burghoff/Scientific-Inkscape). The heatmap shows the negative LOG_{10} of the FDR-corrected p-value obtained in a Fisher test to calculate gene ontology enrichment. Cr: *C. rosea*, Wr: Wheat roots.

Additional File 2: Figure S2

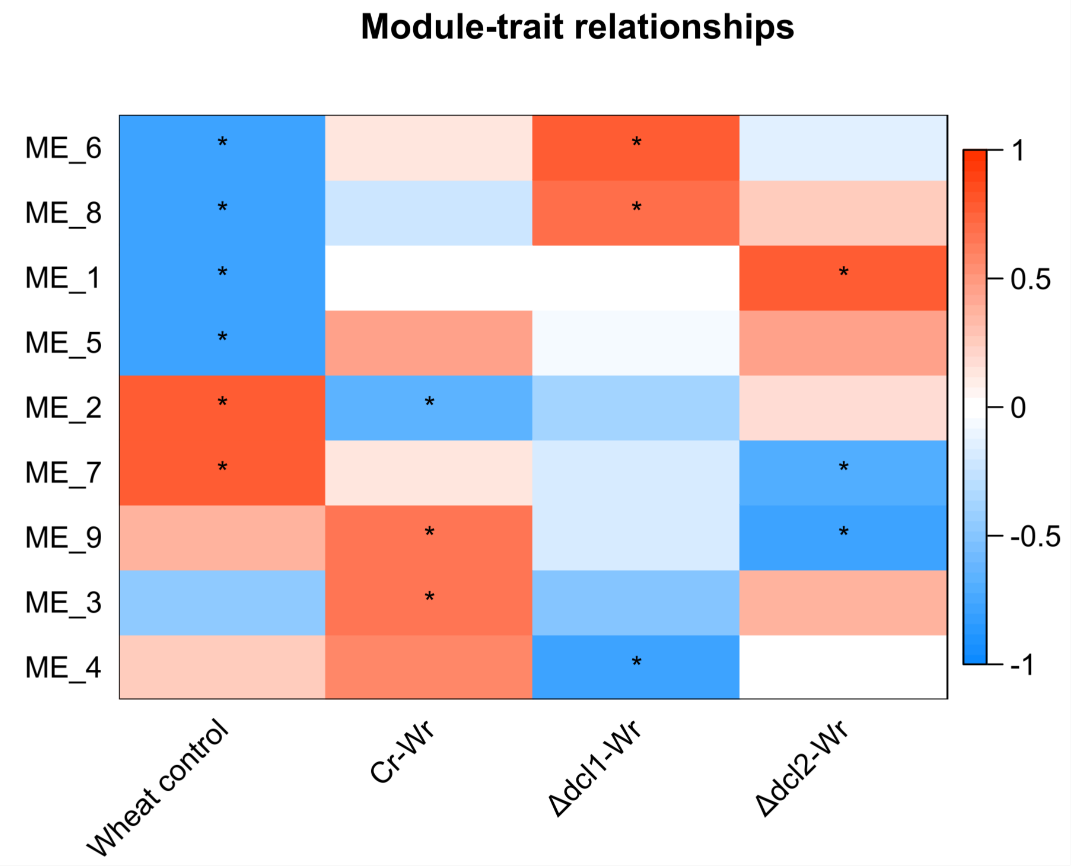

Figure S2: The heatmap shows the Spearman correlation between the module eigengenes of coexpression modules generated with WGCNA and the conditions examined in this study. Wheat roots (Wheat Control), *C. rosea* WT interacting with wheat roots (Cr-Wr), *C. rosea* Δ*dcl1* interacting with wheat roots (Δ*dcl1*-Wr), *C. rosea* Δ*dcl2* interacting with wheat roots (Δ*dcl2*-Wr). The modules were generated using the normalized expression values of differentially expressed wheat genes. Asterisks indicate significant correlation or anticorrelation. Cr: *C. rosea*, Wr: Wheat roots.

Additional File 2: Figure S3

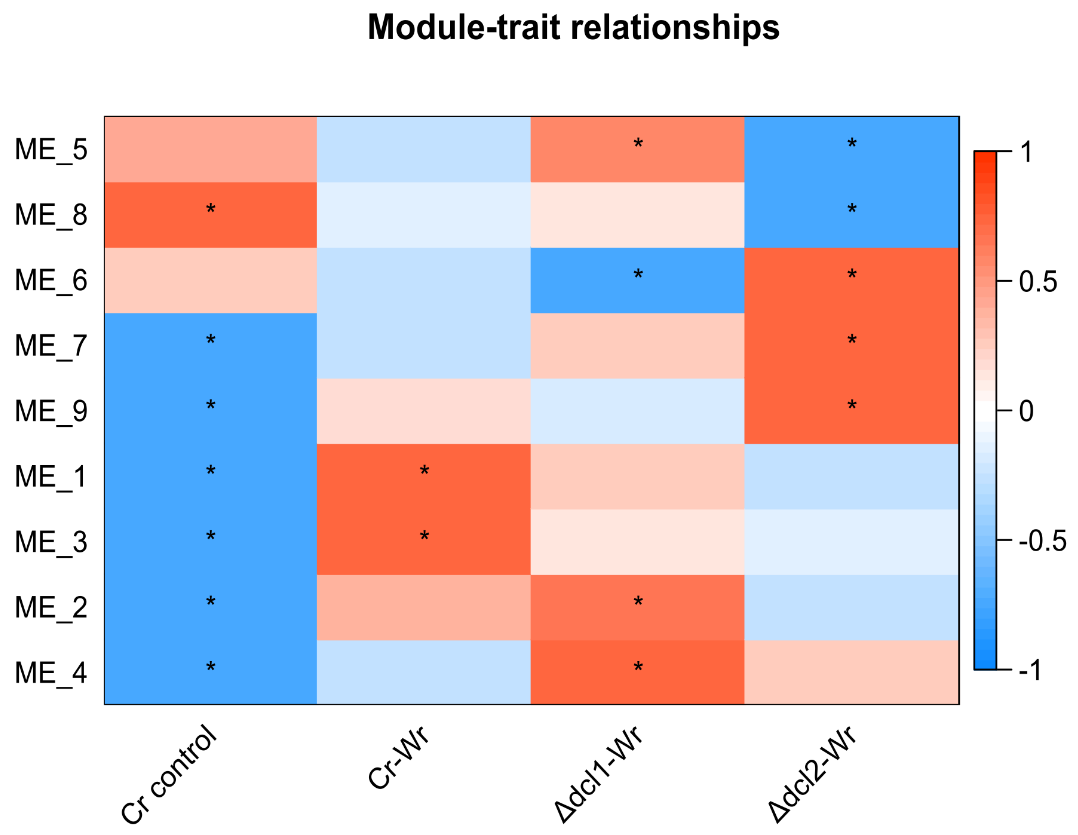

Figure S3: The heatmap shows the Spearman correlation between the module eigengenes of coexpression modules generated with WGCNA and the conditions examined in this study. *C. rosea* WT growing in PDB media (Cr Control), *C. rosea* WT interacting with wheat roots (Cr-Wr), *C. rosea* Δ*dcl1* interacting with wheat roots (Δ*dcl1*-Wr), *C. rosea* Δ*dcl2* interacting with wheat roots (Δ*dcl2*-Wr). The modules were generated using the normalized expression values of differentially expressed *C. rosea* genes. Asterisks indicate significant correlation or anticorrelation. Cr: *C. rosea*, Wr: Wheat roots.

Additional File 2: Figure S4

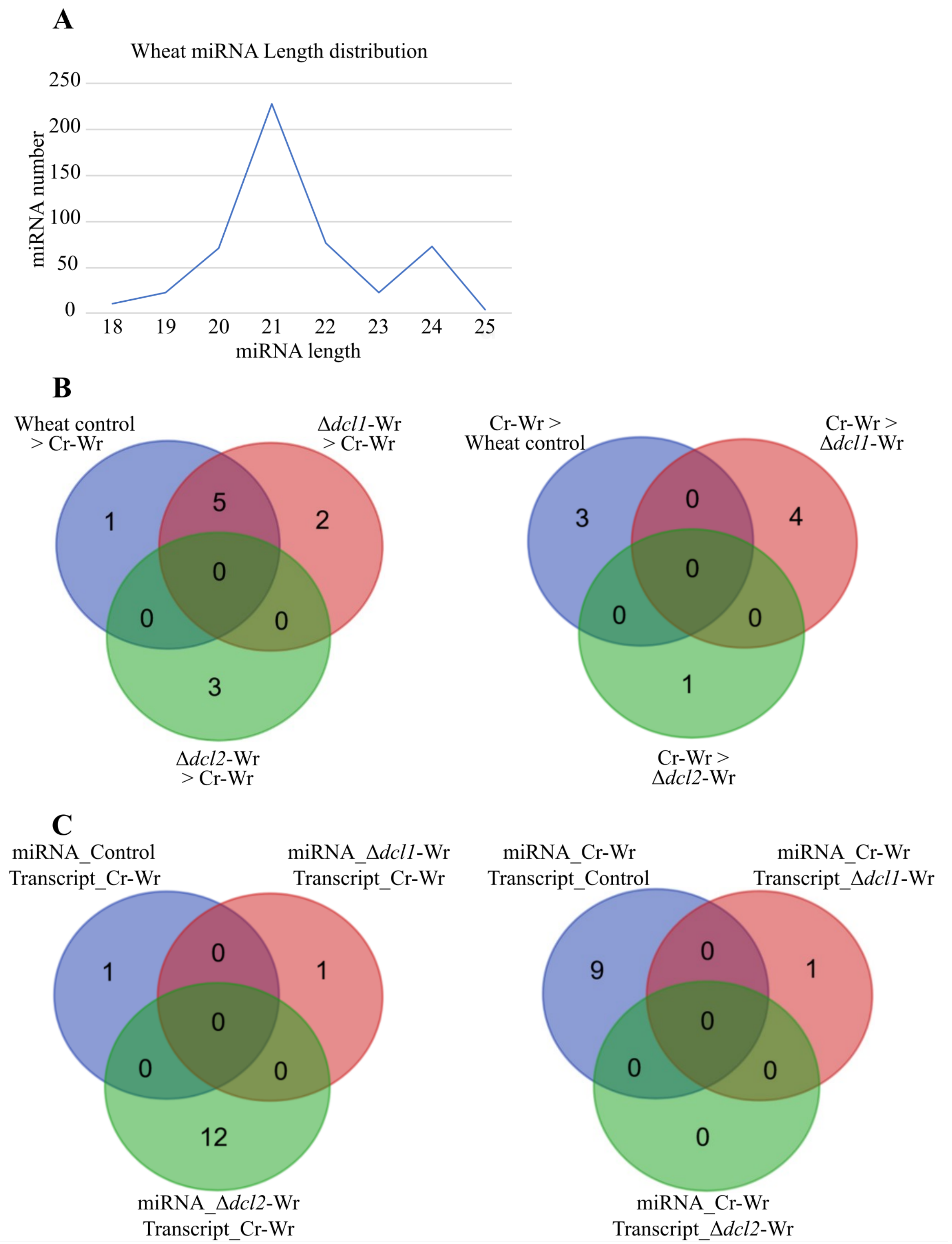

Figure S4: The figure contains information regarding the wheat miRNAs detected in this study. **A**: length distribution. **B**: differential expression. **C**: number of putative gene targets showing inverse expression pattern with the miRNAs.

Additional File 2: Figure S5

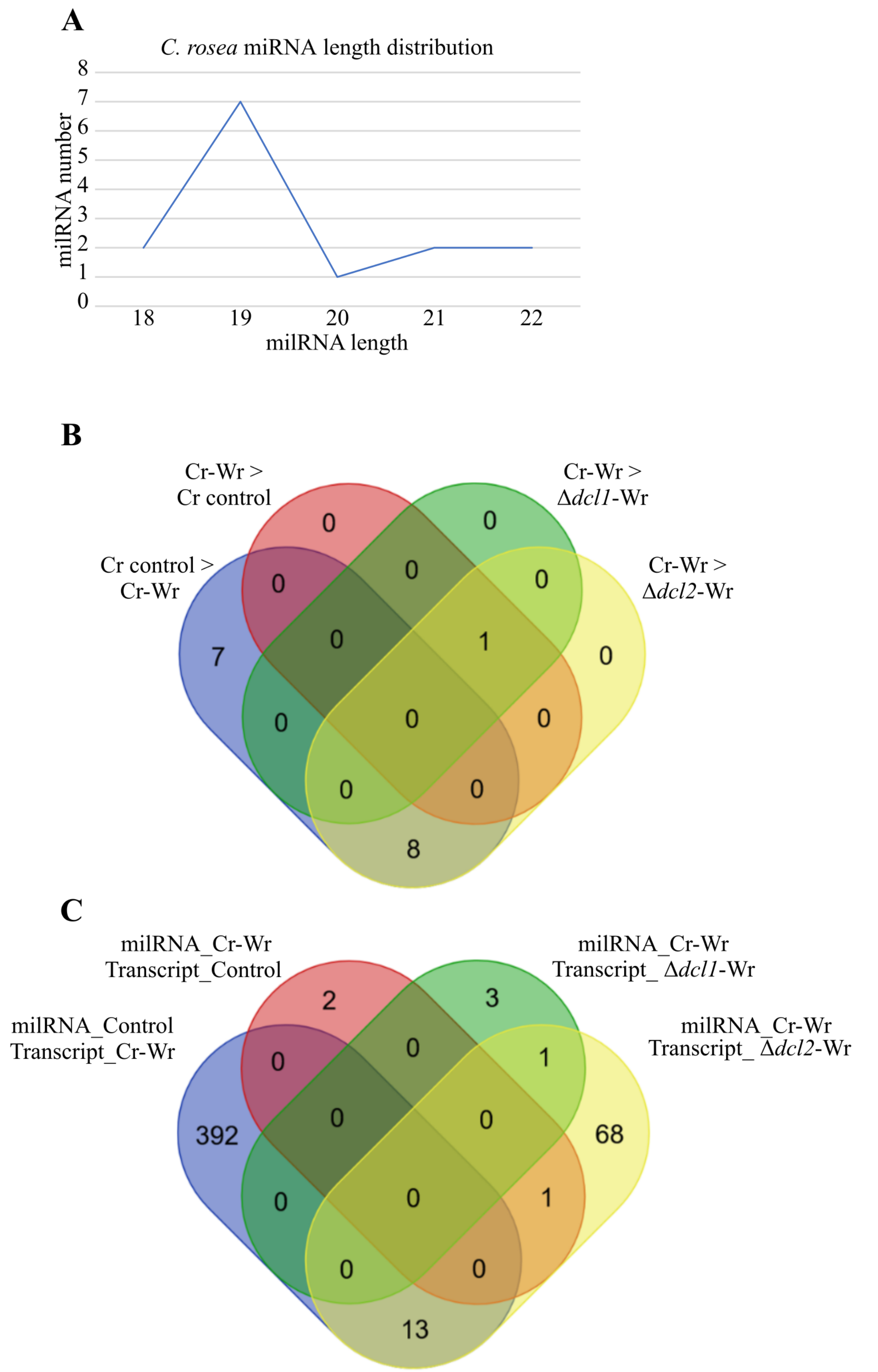

Figure S5: The contains information regarding the *C. rosea* milRNAs detected in this study. **A**: length distribution. **B**: differential expression. C: number of putative gene targets showing an inverse expression pattern with the milRNAs.

Additional File 2: Figure S6

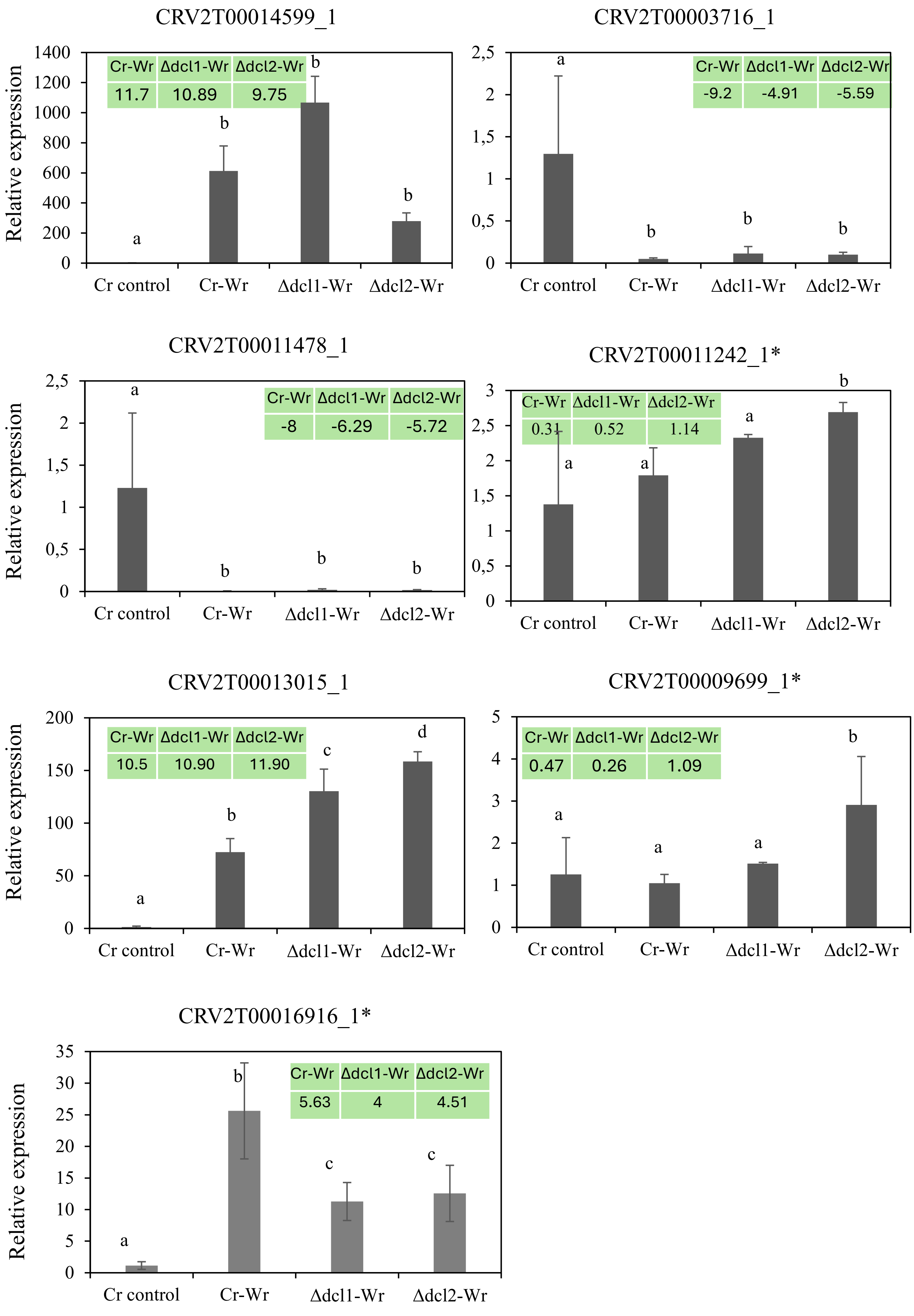

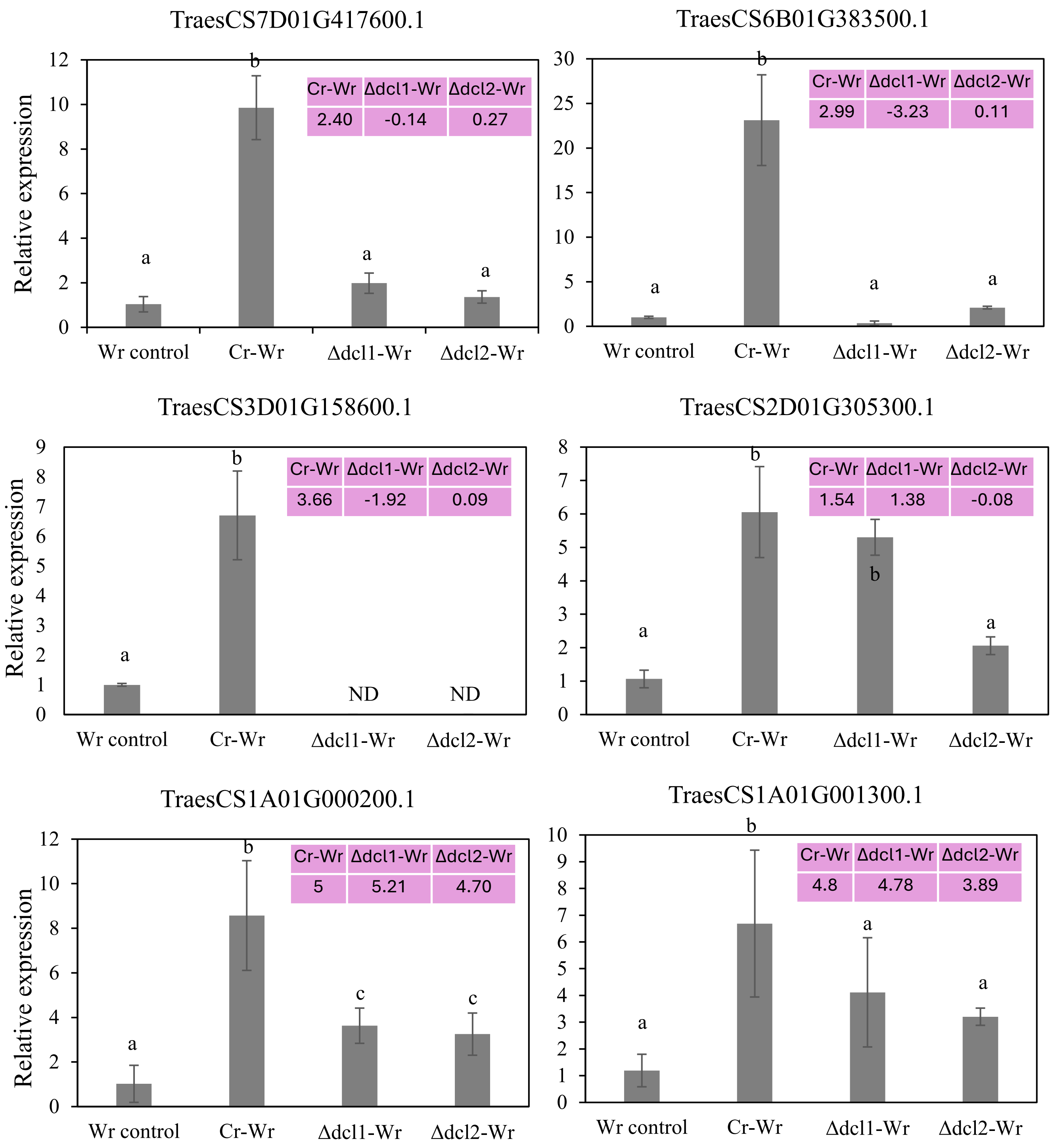

Figure: S6. Gene expression validation by RT-qPCR. Expression profiles of selected *C. rosea* (A) and wheat (B) genes were analyzed during interactions. Relative expression levels in *C. rosea* and wheat were normalized by respective *C. rosea* and wheat β-tubulin (TUB) expression and presented in relation to non-interaction control. Error bars represent standard deviation based on three biological replicates. Different letters indicate statistically significant differences ($P < 0.05$) based on Fisher's exact test. The table highlighted in green indicates gene expression patterns from RNAseq. *indicates endogenous gene targets (*C. rosea* genes targeted by *C. rosea* milRNAs, see table 4), # indicates cross-kingdom gene targets (*C. rosea* gene targeted by three wheat miRNAs mir_17532_x1, mir_16010_x2, mir_12061_x13 (see table 4).

Additional File 2: Figure S7

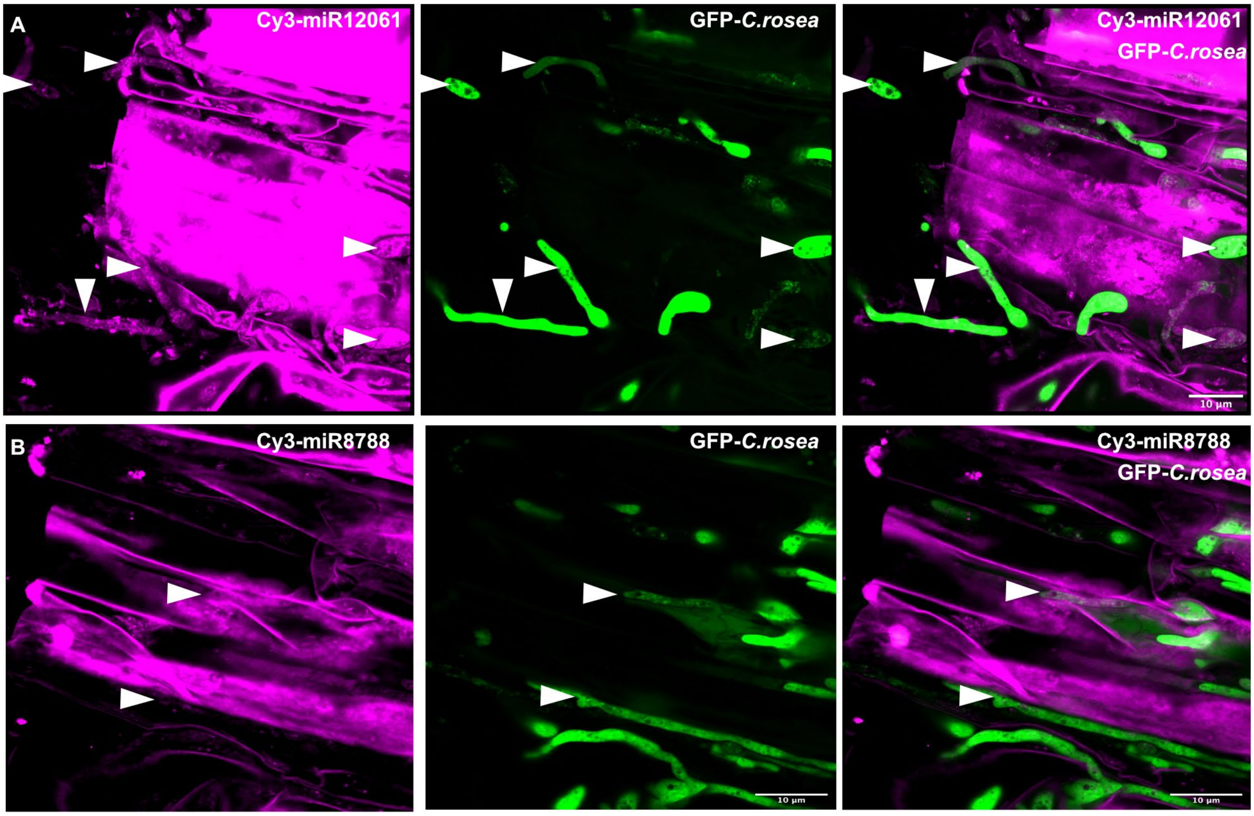

Figure S7: Trafficking of Cy3 labelled wheat mir_12061_x13 mimics (miR17532) (A) and *Phytophthora infestans* miR8788 (B) from wheat roots to *C. rosea*-GFP conidia and hyphae **A.** Representative confocal images showing the co-localization of Cy3-miR17532 (Magenta), *C. rosea*-GFP (Green) and merge (right panel). **B.** Representative confocal images showing the co-localization of Cy3-miR8788 (Magenta), *C. rosea*-GFP (Green) and merge (right panel). Twenty-four hours post incubation (hpi) with the mimics, wheat roots were washed with 0.M KCl and 0.01 M Triton X100 to remove surface-bound miRNA oligos. Conidia from *C. rosea*-GFP were applied to the roots, and Cy3 fluorescence was determined 72 hpi.
